# Interaction of Purinergic P2X4 and P2X7 Receptor Subunits

**DOI:** 10.3389/fphar.2017.00860

**Published:** 2017-11-22

**Authors:** Markus Schneider, Kirsten Prudic, Anja Pippel, Manuela Klapperstück, Ursula Braam, Christa E. Müller, Günther Schmalzing, Fritz Markwardt

**Affiliations:** ^1^Julius-Bernstein-Institute for Physiology, Martin-Luther-University, Halle, Germany; ^2^Molecular Pharmacology, RWTH Aachen University, Aachen, Germany; ^3^Pharmaceutical Institute, Pharmaceutical Chemistry I, University of Bonn, Bonn, Germany

**Keywords:** P2X7, P2X4, voltage clamp, fluorescence, FRET, interaction, subunit

## Abstract

P2X4 and P2X7 are members of the P2X receptor family, comprising seven isoforms (P2X1–P2X7) that form homo- and heterotrimeric non-specific cation channels gated by extracellular ATP. P2X4 and P2X7 are widely coexpressed, particularly in secretory epithelial cells and immune and inflammatory cells, and regulate inflammation and nociception. Although functional heteromerization has been established for P2X2 and P2X3 subunits expressed in sensory neurons, there are contradictory reports regarding a functional interaction between P2X4 and P2X7 subunits. To resolve this issue, we coexpressed P2X4 and P2X7 receptor subunits labeled with green (EGFP) and red (TagRFP) fluorescent proteins in *Xenopus laevis* oocytes and investigated a putative physical interaction between the fusion proteins by Förster resonance energy transfer (FRET). Coexpression of P2X4 and P2X7 subunits with EGFP and TagRFP located in the extracellular receptor domains led to significant FRET signals. Significant FRET signals were also measured between C-terminally fluorophore-labeled full-length P2X4^1-384^ and C-terminally truncated fluorescent P2X7^1-408^ subunits. We furthermore used the two-electrode voltage clamp technique to investigate whether human P2X4 and P2X7 receptors (hP2X4, hP2X7) functionally interact at the level of ATP-induced whole-cell currents. Concentration–response curves and effects of ivermectin (P2X4-potentiating drug) or BzATP (P2X7-specific agonist) were consistent with a model in which coexpressed hP2X4 and hP2X7 do not interact. Similarly, the effect of adding specific inhibitors of P2X4 (PSB-15417) or P2X7 (oATP, A438079) could be explained by a model in which only homomers exist, and that these are blocked by the respective antagonist. In conclusion, we show that P2X4 and P2X7 subunits can form heterotrimeric P2X4/P2X7 receptors. However, unlike observations for P2X2 and P2X3, coexpression of P2X4 and P2X7 subunits does not result in a novel electrophysiologically discriminable P2X receptor phenotype.

## Introduction

P2X receptors are non-selective cation channels that are opened by extracellular ATP. The presence of ATP is considered a danger-associated signal (DAMP, danger-associated molecular pattern), because ATP is released from cells during damage, hypoxia, or cell membrane stretching (Di Virgilio, 2007; Praetorius and Leipziger, 2009). P2X receptors are homo- or heterotrimers that assemble from a repertoire of seven possible subunits (P2X1–7) (Nicke et al., 1998; Aschrafi et al., 2004). All P2X subunits share similar membrane topology: intracellular N- and C-termini, two membrane-spanning domains (TM1 and TM2), and a large extracellular loop comprising the ATP binding site. The TM2 domains form the gate and the selectivity filter (Baconguis et al., 2013; Habermacher et al., 2015; Pippel et al., 2017). In addition to homotrimeric P2X receptors, P2X subunits can form heteromeric ion channels. The existence of P2X2/P2X3 heteromers in sensory neurons is well established (Lewis et al., 1995). A large number of other heteromers have been characterized in heterologous expression systems. However, their function in native tissue remains to be established (Saul et al., 2013).

The P2X4 and P2X7 subunit isoforms are widely coexpressed, particularly in secretory epithelial cells and cells of the immune and inflammatory system. The P2X7 subunit has the highest amino acid sequence similarity to the P2X4 subtype (Burnstock and Knight, 2004; Surprenant and North, 2008). Furthermore, the genes encoding both of these subunits colocalize on human chromosome 12, where they are separated by only 24 kbp (Craigie et al., 2013). P2X7 mRNA levels are reduced in P2X4 knockout mice, and vice versa (Craigie et al., 2013). In contrast, shRNA-mediated downregulation of P2X7 mRNA leads to increased P2X4 expression. Knockdown of P2X4 mRNA is reported to result in a compensatory increase in P2X7 protein expression (Weinhold et al., 2010).

Early co-immunoprecipitation studies of P2X subunit interactions excluded the interaction of P2X7 subunits with other subtypes (Torres et al., 1999), although later studies reported evidence of P2X4/P2X7 heteromerization (Guo et al., 2007). Subsequent investigations, however, have questioned the existence of P2X4/P2X7 heteromeric receptors (Nicke, 2008; Antonio et al., 2011; for reviews, see Craigie et al., 2013; Saul et al., 2013).

Here, we investigated physical and functional interactions P2X4 and P2X7 subunits heterologously expressed in *Xenopus* oocytes by measuring P2X4/P2X7-dependent Förster (or fluorescence) resonance energy transfer (FRET) signals and ion currents in *Xenopus* oocytes.

## Materials and Methods

### Reagents

Unless otherwise stated, we purchased chemicals and molecular biology reagents from Sigma–Aldrich (Taufkirchen, Germany), Merck (Darmstadt, Germany), and New England Biolabs (Schwalbach, Germany). The novel hP2X4-selective antagonist PSB-15417 was provided by Prof. Christa Müller (Institute of Pharmaceutical Chemistry, University of Bonn, Germany) via Orion (Espoo, Finland).

### Expression of hP2X4 and hP2X7 Subunits in *Xenopus laevis* Oocytes

The following oocyte expression plasmids encoding full-length human (h) and rat (r) subunits of ligand-gated ion channels were available from our previous work (reference sequence NCBI IDs and references in parenthesis): hP2X4 (ID: NP_002551.2, Rettinger et al., 2000); rP2X4 (ID: NP_113782.1, Aschrafi et al., 2004); hP2X7 (ID: NP_002553.3, Klapperstück et al., 2000), hP2X7^1-408^ (C-terminally truncated by placing a premature TGA stop codon directly after the hP2X7 ^408^H codon, Becker et al., 2008); and hGLYRA1 (ID: NP_000162.2, Büttner et al., 2001). We amplified full-length cDNA encoding the rat P2X7 subunit (ID: NP_062129.1) by RT-PCR from total rat brain RNA isolated using the RNA Clean System (Angewandte Gentechnologie Systeme, Heidelberg, Germany) and gene-specific primers (Supplementary Table 1) based on the published rP2X7 sequence (Surprenant et al., 1996). The PCR product was first inserted into the pGEM5 ZF(+) vector (X65308; Promega, Mannheim, Germany) by TA cloning (Kovalic et al., 1991) and then directionally subcloned it into the pNKS2 oocyte expression vector (Gloor et al., 1995) using *Aat*II and *Xba*I restriction sites introduced via PCR (underlined in Supplementary Table 1). We previously reported using the rP2X7-pNKS2 construct without describing its origin (Hausmann et al., 2006).

We obtained a synthetic gene encoding the *Caenorhabditis elegans* glutamate-gated chloride channel α (GluCl) optimized for crystallization (GluCl_cryst_) (Hibbs and Gouaux, 2011) from ShineGene (Shanghai, China). This was subcloned into a Gateway-compatible pNKS2 vector (Stolz et al., 2015) using the Gateway cloning system (Invitrogen, Karlsruhe, Germany). We previously verified by blue native PAGE that ectopic GluCl_cryst_ efficiently assembles into a homopentamer in *Xenopus laevis* oocytes (Dopychai et al., 2015). A plasmid harboring full-length cDNA for hTRPV2 (DNASU plasmid ID HsCD00045624) was obtained from the DNASU Plasmid Repository (The Biodesign Institute, Arizona State University, Tempe, AZ, United States) and subcloned using the Gateway system into the pNKS2 vector.

We generated fluorophore-labeled channel constructs with the enhanced green fluorescent protein or Tag red fluorescent protein (referred to as GFP or RFP throughout, respectively) located at the N-terminus (or ectodomain) or C-terminus (indicated by adding the name of the label (GFP or RFP) at the left (ectodomain) or right (C-terminus) of the fusion protein name). To N-terminally labeling hGLYRA1 (the human glycine receptor α1 subunit) with GFP, we first located the signal peptidase cleavage site at between codon position ^28^A and ^29^A using the SignalP 4.1 server^1^ (Petersen et al., 2011). Next, we introduced unique *Nde*I and EcoR47III restriction sites using the QuikChange site-directed mutagenesis protocol (Stratagene, Heidelberg, Germany, (Braman et al., 1996) with primers O-1699/O-1700 (Supplementary Table 2). Finally, we PCR-amplified the full-length cDNA for GFP from the EGFP-N1 vector (Invitrogen, Karlsruhe, Germany) using primers O-1701/O-1702 (Supplementary Table 3), purified, and directionally cloned between *Nde*I and EcoR47III sites to construct the SP-GFP-hGLYRA1 vector (where SP indicates the position of the signal peptide). This construct encodes an additional alanine residue directly 5′ of the GFP moiety and is predicted by the SignalP 4.1 server to undergo signal peptidase cleavage at the same residues as wild type (wt) hGLYRA1.

We used the megaprimer method (Perez et al., 2006) for fluorescence labeling of all the other ion channel constructs (primers are listed in Supplementary Table 2). Single (GFP or RFP) or tandem (GFP-RFP) labels were added to the C-termini of ion channel constructs by in-frame fusion with full-length GFP and RFP sequences with the RFP sequence amplified from the TagRFP-N vector (Evrogen, Moscow, Russia). Likewise, we inserted GFP and RFP cDNA either singly or in tandem after *P2X4* codon 122 or *P2X7* codon 125. Our rational was that rP2X4 receptors containing a fluorescent pHluorin moiety after ^122^K have previously been shown to function like wt-rP2X4 (Xu et al., 2014). A previous sequence alignment showed that rP2X4 ^122^K (hP2X4 ^122^A) corresponds to ^125^R for both rP2X7 and hP2X7 (Kawate et al., 2009).

We synthesized capped cRNA using a modified method (Klapperstück et al., 2000) involving co-transcriptional incorporation of the anti-reverse cap analog (m_2_^7,3′-O^GpppG; NU-855; Jena Bioscience, Germany) to ensure the correct orientation at the ATG start codon of the cRNA (Grudzien-Nogalska et al., 2007; Stolz et al., 2015). We surgically isolated oocytes from tricaine-anesthetized *Xenopus laevis* (Xenopus Express, Vernassal, France) using sterile surgical techniques and defolliculated them with collagenase NB 4G (Serva, Heidelberg, Germany). We injected oocytes of Dumont stages V–VI individually with 5–50 ng *P2X4* and/or *P2X7* cRNA to obtain similar ATP-evoked current amplitudes mediated by the encoded P2X4 and P2X7 receptors. To optimize FRET efficiency (FE), we adjusted the amount of mRNA used to coexpress GFP- and RFP- labeled constructs to obtain higher fluorescence signals from channels labeled with GFP than with RFP (Ma et al., 2014). We incubated the oocytes at 19°C in sterilized frog Ringer solution (Mg/Ca-ORi: 100 mM NaCl, 2.5 mM KCl, 1 mM MgCl_2_, 1 mM CaCl_2_, and 10 mM HEPES, pH 7.4) supplemented with penicillin (100 U/ml) and streptomycin (100 μg/ml) or with gentamycin (50 μg/ml) (Flittiger et al., 2010). This study was carried out in accordance with the recommendations of the EC Directive 86/609/EEC for animal experimentation. The protocol was approved by the local animal welfare committee (reference no. 53a-42502/2–173; Magdeburg, Germany).

### FRET Experiments

We measured fluorescence signals from human and rat P2X4 and P2X7 subunits labeled with GFP as the donor (excitation at 488 nm, emission at 500–550 nm) or RFP as the acceptor (excitation at 561 nm, emission at 570–650 nm) using a TCS SP5 spectral confocal laser scanning microscope (Leica Microsystems, Wetzlar, Germany) with a 20 × 0.5 HC PL Fluotar dry objective lens. We placed individual oocytes expressing the indicated constructs in a IBIDI μ-Slide 8-well chamber in oocyte Ringer solution (ORi: 100 mM NaCl, 2.5 mM KCl, 1 mM MgCl_2_, 1 mM CaCl_2_, and 5 mM HEPES, pH 7.4) and measured fluorescence signals from the oocyte pole adjacent to the chamber bottom or at the oocyte equator (which had similar FEs; **Figure 2**). Fluorescence signals from donor GFP and acceptor RFP labels were measured before and after acceptor photobleaching at 561 nm using Leica TCS SP5 software or FIJI^2^.

### Two-Electrode Voltage Clamp Electrophysiology

We performed the electrophysiological experiments at room temperature (∼22°C) as previously described (Stolz et al., 2015). We accomplished rapid, reproducible solution exchange by using a small tube-like chamber (0.1 ml) combined with fast superfusion (75 μl/s). A set of computer-controlled magnetic valves combined with a modified U-tube technique permitted the bathing solutions to be changed in less than 1 s (Klapperstück et al., 2000). We recorded whole-cell membrane currents via the two-electrode voltage clamp method using 3 M KCl-filled microelectrodes (resistance range 0.8–1.2 MΩ). The currents were recorded and filtered at 100 Hz using an oocyte clamp amplifier (OC-725C; Hamden, CT, United States) and sampled at 85 Hz. We stored and analyzed the data on a personal computer using software written in our department (Superpatch 2000, SP-Analyzer by T. Böhm). Two or three days after cRNA injection, we superfused individual oocytes with ORi solution and impaled the voltage clamp electrodes. To measure currents induced by ATP^4-^, the agonist of the P2X4 and P2X7 receptors (Klapperstück et al., 2001; Li et al., 2013), we switched the bathing solution to Ca^2+^- and Mg^2+^-free ORi (ORi0Ca0Mg: 100 mM NaCl, 2.5 mM KCl, 5 mM HEPES, and 0.1 mM flufenamic acid, pH 7.4) to prevent metal ion complexation of ATP^4-^. We included flufenamic acid in the ORi0Ca0Mg solution to suppress the large inward conductance that develops in the absence of divalent ions (Kubick et al., 2011). Agonists and antagonists were diluted in the ORi0Ca0Mg solution, as indicated in the figures.

### Data Analysis and Presentation

For approximations, statistical analysis, and presentation of the data we used SigmaPlot software (Systat Software). Statistical data were analyzed using one-way ANOVA. We tested the statistical significance of the differences between means using the Bonferroni multiple comparison *t*-test. The significance of the correlation coefficients was tested using the *t*-test. Statistical significance was set at *p*-value of <0.05.

## Results

### FRET Demonstrates a Physical and Functional Interaction between P2X4 and P2X7

**FIGURE 1:**
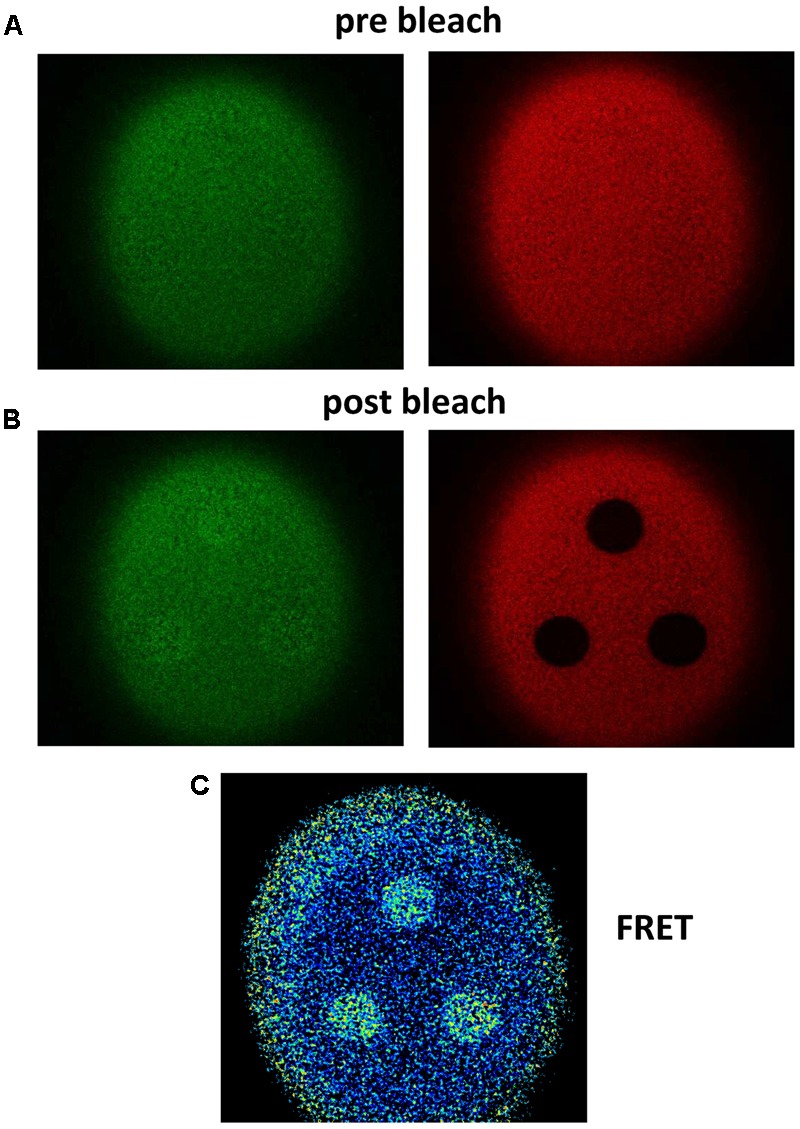
Fluorescence signals before and after acceptor photobleaching. Example of Donor (GFP) recovery after acceptor (RFP) photobleaching for the P2X4 construct with a C-terminal tandem GFP-RFP label. **(A,B)** Pre-bleaching **(A)** and post-bleaching **(B)** fluorescence images were obtained as described in the Methods section using a confocal laser scanning microscope. The slightly increasing GFP fluorescence is shown on the left side and the RFP fluorescence, which is strongly decreased due to application of the maximal RFP exciting light for 1 min on the right side. **(C)** The FRET signal was calculated pointwise using the microscope software in the same manner as shown in **Figure 2**. A strong FRET signal is measured in areas of RFP bleaching. Blue pixels indicate low and yellow pixels high FE.

**FIGURE 2:**
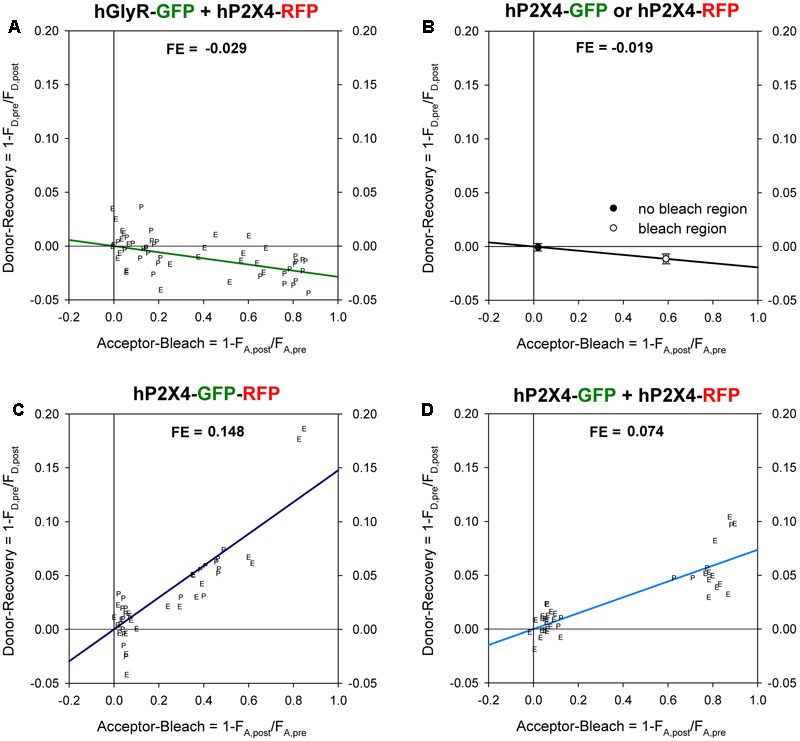
Förster resonance energy transfer (FRET) between GFP- and RFP -labeled P2XR subunits measured with donor recovery by acceptor photobleaching. Measurements were made at the oocyte pole adjacent to the bottom of the recording chamber (P) or at the equator (E) of the oocytes, as shown in **Figure 1**. The degree of donor recovery was plotted against the degree of acceptor bleaching. FRET efficiency (FE) was obtained by extrapolating the linear regression line to complete acceptor bleaching (right hand *y*-axis). **(A)** Negative control: coexpression of C-terminally RFP-labeled hP2X4 with SP-GFP-GLYRA1. **(B)** In oocytes expressing either hP2X4-GFP or hP2X4-RFP, the mean changes in the donor (donor recovery) and acceptor fluorescence (acceptor bleach) were measured at bleached versus non-bleached areas. The decrease of the donor fluorescence in regions of acceptor bleach indicates a bleaching effect of the acceptor excitation light and leads to calculation of negative FE values. **(C)** Positive control: P2X4 protein with a C-terminal tandem GFP-RFP label. **(D)** Coexpression of C-terminally GFP- and RFP-labeled hP2X4 constructs. Mean fluorescence values were calculated from 50–60 regions of interests (ROIs) in 5–10 oocytes.

To investigate whether heteromerization occurs between P2X4 and P2X7 receptor subunits, we used the donor dequenching after acceptor photobleaching FRET quantification technique (Zheng, 2006; Ma et al., 2014). GFP- or RFP-dependent fluorescence was measured by laser scanning fluorimetry at the oocyte equator or at the oocyte pole in contact with the bottom of the recording chamber before (**Figure 1A**) and after (**Figure 1B**) photobleaching of the RFP-labeled acceptor construct. The FRET efficiency was visualized using the microscope software (**Figure 1C**) following quantitative evaluation (as demonstrated in **Figure 2**). The percentage increase in GFP fluorescence at different bleaching regions was calculated using Equation 1:

$$\mathrm{Donor}\;\mathrm{recovery}=1-F_{D,\mathrm{pre}}/F_{D,\mathrm{post}}$$

where F_D,pre_ and F_D,post_ are the mean fluorescence signals pre- and post-bleaching, respectively, for the donor GFP at bleached regions. The percentage increase in GFP fluorescence was plotted against the percentage decrease in RFP fluorescence, as calculated using Equation 2:

$$\mathrm{Acceptor}\;\mathrm{bleach}=1-F_{A,\mathrm{post}}/F_{A,\mathrm{pre}}$$

where F_A,pre_ and F_A,post_ are the mean pre- and post-bleaching fluorescence signals of the acceptor RFP. Extrapolation to 100% acceptor bleaching yielded the FE (Nashmi et al., 2003). FE values measured at the oocyte pole adjacent to the recording chamber were not significantly different from those measured at the oocyte equator (marked “P” and “E”, respectively, in **Figure 2**).

Negative controls had negative FE values (**Figure 2A**) due to the reduction in GFP fluorescence during acceptor photobleaching (**Figure 2B**). A maximal FRET efficiency of 0.148 was measured in oocytes expressing the P2X4-GFP-RFP tandem by extrapolation of the regression line shown in **Figure 2C** to 1.0. A representative example of measurements of a positive control (i.e., oocytes coexpressing P2X4-GFP and P2X4-RFP subunits) is shown in **Figure 2D**.

**FIGURE 3:**
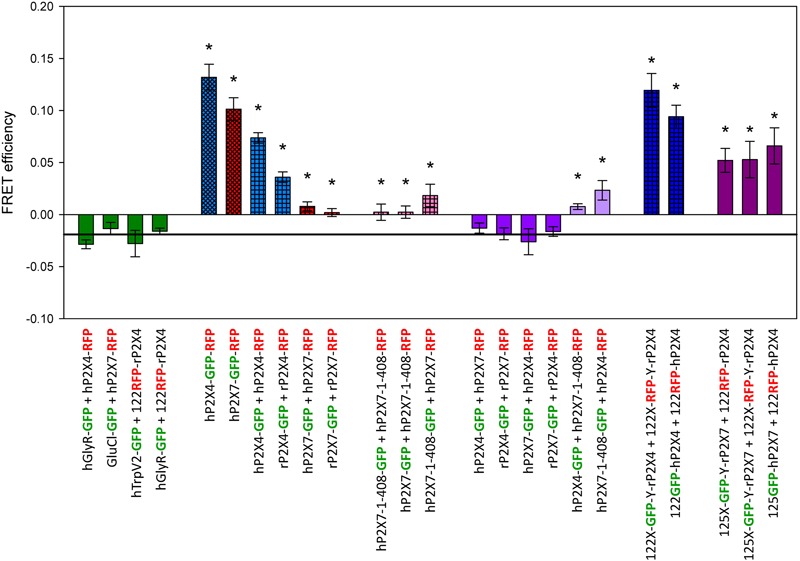
Förster resonance energy transfer measurement of P2X4 and P2X7 subunit interactions. FRET efficiencies were calculated as described in **Figure 2**. Horizontal line represents GFP bleaching during acceptor bleaching leading to negative FE values of –0.019 (see **Figure 2B**). ^∗^, FRET efficiencies significantly larger than –0.019. Data are the means ± SEM from 12–20 oocytes. X and Y denote short, flexible linkers (-GAGA- and -AGAG-, respectively; single amino acid letter code) flanking the GFP or RFP moiety. The position of the fluorophore name indicates the position of the label in the P2X receptor fusion construct (i.e., P2X7-GFP indicates a C-terminal label). Bar colors: green = negative control, blue = P2X4 constructs, red = P2X7 constructs, pink = P2X4/P2X7 coexpression. Bar patterns: cross-hatched = tandem GFP-RFP label, checkered = positive controls.

Mean FRET measurements for different P2X4 and P2X7 constructs labeled with GFP or RFP are depicted in **Figure 3**. As negative controls, we coexpressed RFP-labeled P2X4 or P2X7 constructs with the tetrameric hTRPV2 channel, the pentameric human glycine receptor α1 hGLYRA1, or the pentameric invertebrate glutamate-gated chloride channel GluCl. FRET values for each of these combinations did not significantly differ from –0.019 (determined for the negative control example shown in **Figure 1**). The largest FRET signals were measured for the C-terminal tandemly labeled proteins hP2X4-GFP-RFP and hP2X7-GFP-RFP, and for the P2X4 constructs with single fluorescent labels in the extracellular domain (^122^GFP-P2X4 + ^122^RFP-P2X4). Robust FRET signals were also measured after coexpressing the C-terminally labeled positive controls P2X4-GFP and P2X4-RFP. Coexpression of P2X4-GFP with full-length P2X7-RFP did not result in significant FRET signals, but a significant FRET signal was obtained when C-terminally labeled truncated P2X7^1-408^ and P2X4 were coexpressed. These values were similar to the FRET efficiencies measured after coexpression of C-terminally GFP- or RFP-labeled truncated hP2X7^1-408^ with full-length P2X7-RFP or P2X7-GFP subunits, respectively. We consider these combinations as quasi-positive controls because we previously showed that the truncated hP2X7 can co-assemble (i.e., physically interact) and function together with full-length hP2X7 receptor subunits (Becker et al., 2008). Much larger FRET signals were obtained for coexpressed P2X4 constructs with GFP or RFP located within the extracellular domain. Similarly, relatively higher FRET values were measured for P2X7 subunits with the GFP label located in the extracellular domain when coexpressed with P2X4 subunits with an extracellularly located RFP label. These results clearly indicate a close physical interaction between P2X4 and P2X7 subunits.

Next, we tested whether the significant FE values between GFP- and RFP-labeled subunits were due to non-specific FRET, i.e., interaction of homotrimeric P2X4 and P2X7 receptors. In this case, the FE should increase with higher levels of protein expression. However, the FE did not correlate with the strength of the fluorescence signal (Supplementary Figure 1).

**FIGURE 4:**
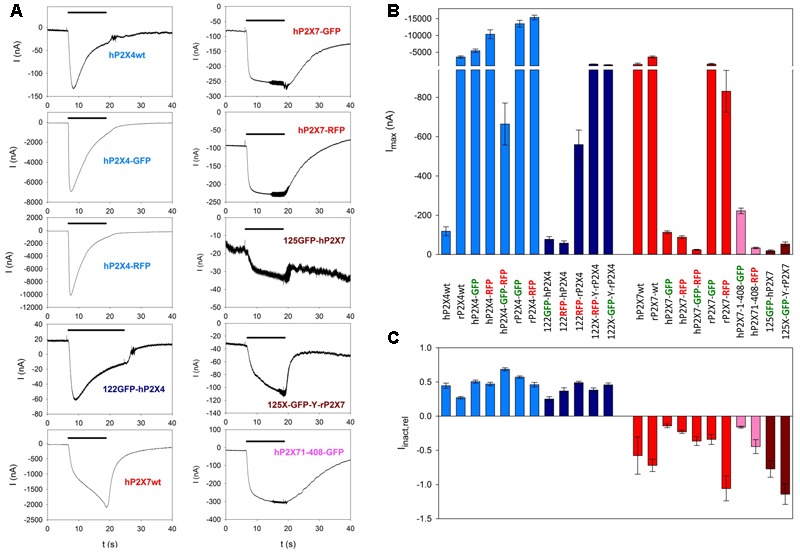
Functional testing of fluorescence-labeled P2X4 and P2X7 constructs by two-electrode voltage clamp measurements. **(A)** Examples of current traces in oocytes expressing wildtype P2X4 (P2X4wt) of P2X7 (P2X7wt) or different fluorescence-labeled P2X4 and P2X7 constructs, as indicated. The colors of the construct names relate to the colors of the bars in **(B,C)**. **(B)** Maximal current amplitudes obtained during the application of 1 mM ATP for 6 s. **(C)** Kinetics of ion channel currents in oocytes expressing different labeled P2X constructs. The relative inactivation (I_inact,rel_) was calculated as the amplitude of the current between 2 and 6 s after the application of 1 mM ATP normalized to the current amplitude at the 2 s time point. A decay in the current amplitude (I_inact,rel_ > 0) indicates desensitization (seen for all P2X4 constructs); an increase in current amplitude during this period (I_inact,rel_ < 0) was usually seen for P2X7 constructs and is of unknown origin. Bar colors: light blue = P2X4wt or C-terminally labeled constructs, dark blue = P2X4 constructs labeled within the extracelllar domain, red = P2X7wt or C-terminally labeled constructs, rosy = C-terminally truncated and labeled P2X7 constructs, dark red = P2X7 constructs labeled within the extracelllar domain. Data are the means ± SEM from 5–20 oocytes.

Fluorophore-labeled P2X constructs formed functional ion channels (shown in **Figure 4**). Although the current amplitudes varied considerably depending on the presence and location of the fluorescent label (**Figures 4A,B**), the characteristics of P2X4-dependent (inactivating) and P2X7-dependent (slowly increasing) currents were retained (**Figures 4A,C**).

### The Physical P2X4/P2X7 Interaction Is Not Associated with a Novel Functional Phenotype

#### Kinetics of Coexpressed hP2X4 and hP2X7 Receptor Subunits

**FIGURE 5:**
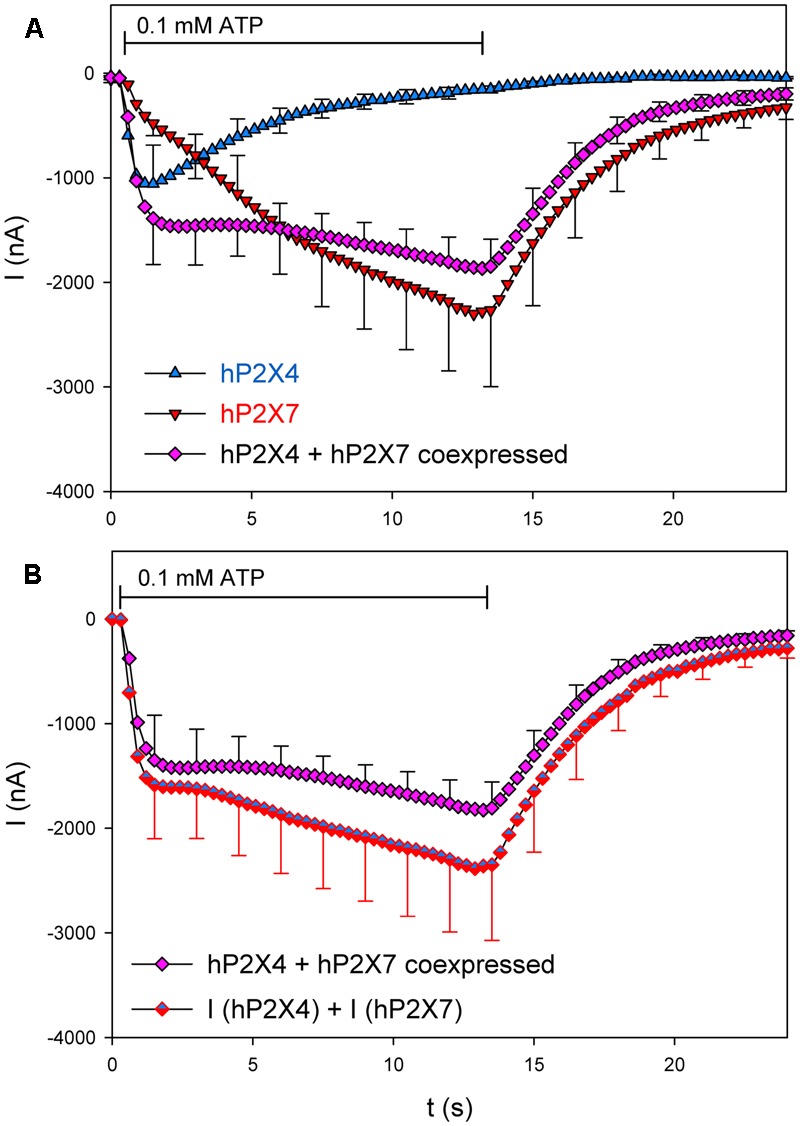
Kinetics of ion channel currents in hP2X4/hP2X7-coexpressing oocytes. Two-electrode voltage clamp measurements of currents in *Xenopus* oocytes expressing hP2X4 and hP2X7 receptors were elicited by the application of 0.1 mM ATP. **(A)** Mean current traces were recorded from oocytes expressing hP2X4 or hP2X7 subunits alone or together, as indicated. **(B)** Mean currents in oocytes coexpressing hP2X4 and hP2X7 were compared with the sum of those in oocytes expressing hP2X4 or hP2X7 alone. The mean current amplitudes were not significantly different at any time point, indicating that P2X4/P2X7 heteromers with distinct current kinetics are not formed. Data are the means ± SD from five oocytes at each time point.

We first tested whether coexpression changes the kinetics of hP2X4- and hP2X7-dependention channel currents. We therefore compared the mean ATP-induced currents in oocytes expressing hP2X4 and/or hP2X7 (**Figure 5A**). hP2X4-dependent currents displayed the typical inactivating behavior, leading to a peak current at about 1 s after ATP application. In contrast, currents in hP2X7-expressing oocytes showed the typical slowly activating non-saturating behavior during the 12 s of ATP application (Klapperstück et al., 2001; Stolz et al., 2015). In hP2X4/hP2X7-coexpressing oocytes, a peak ATP-induced current was barely detectable, and the slope of the slowly activating current was reduced compared with hP2X7-dependent currents. In **Figure 5B**, the current measured in hP2X4 and hP2X7 coexpressing oocytes is compared with the sum of the currents measured in oocytes expressing each protein alone. The Current amplitudes were not significantly different throughout the entire time course of ATP application and withdrawal. The simplest explanation for this observation is that hP2X4 and hP2X7 do not functionally interact in oocytes, and that the measured ATP-dependent current is simply the sum of the separate hP2X4- and hP2X7-dependent components.

#### ATP Dependence of Coexpressed hP2X4 and hP2X7 Receptor Subunits

**FIGURE 6:**
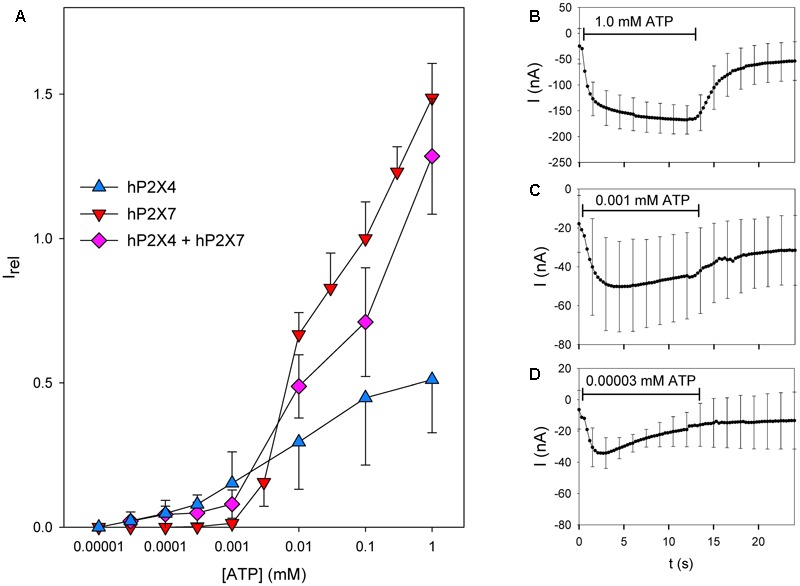
Dependence on the ATP concentration of coexpressed hP2X4 and hP2X7 subunits. **(A)** Maximal current amplitudes in the presence of various ATP concentrations ([ATP]) were obtained by two-electrode voltage clamp measurements in oocytes expressing hP2X4 and/or hP2X7, as indicated. These amplitudes were measured during a 12 s lasting ATP application and normalized to the amplitude during a preceding application of 0.1 mM ATP to yield the relative current amplitude I_rel_. I_rel_ values for hP2X4- or hP2X7-expressing oocytes are significantly different at all ATP concentrations. Current amplitudes of oocytes expressing hP2X4 + hP2X7 are significantly different from those of P2X7-expressing oocytes at 0.1–1 μM ATP and significantly different from those of P2X4-expressing oocytes at 0.01–1 mM ATP. Thus, the ATP concentration–response curve for hP2X4/hP2X7-coexpressing oocytes is dominated by the hP2X4 component at low ATP concentrations and by the P2X7 component at high ATP concentrations, arguing against a distinct P2X4/P2X7 phenotype regarding the agonist sensitivity. **(B–D)** Averaged current traces of different oocytes expressing hP2X4 and hP2X7, elicited by the second ATP application at the indicated concentrations. Data are the means ± SD from 4–10 oocytes.

We next investigated the ATP concentration dependency of the current in oocytes coexpressing hP2X4 and hP2X7 subunits (**Figure 6A**). As P2X4-dependent currents show long-lasting desensitization following ATP application, we normalized all maximal current amplitudes to the maximal current amplitude measured during a foregoing application of 0.1 mM ATP. The concentration–response curve for oocytes coexpressing hP2X4 and hP2X7 was located between the curves for those expressing hP2X4 or hP2X7 alone, indicating that hP2X4 is dominant at low ATP concentrations and hP2X7 is dominant at high ATP concentrations. At low ATP concentrations, the relative currents measured in oocytes coexpressing hP2X4 and hP2X7 were smaller than those in oocytes expressing hP2X4 only. This results from normalization to the current amplitudes evoked by 0.1 mM ATP. This ATP concentration evokes both hP2X4- and hP2X7-dependent currents, leading to a smaller relative current at ATP concentrations of 1–10 μM, although these are predominantly mediated by hP2X4. Similarly, at high ATP concentrations, the relative currents measured in hP2X4/hP2X7-coexpressing oocytes were smaller than those measured in oocytes expressing hP2X7 only. This also results from normalization to the first application of 0.1 mM ATP, where the hP2X4 component contributes. The hP2X4 component largely undergoes desensitization at the second ATP application, as shown in **Figures 6B–D**, where the mean currents measured in hP2X4/hP2X7-coexpressing oocytes at different ATP concentrations are displayed. At high ATP concentrations (**Figure 6B**), a slowly activating current, driven by the dominant hP2X7 component, was measured. In contrast, at very low ATP concentrations (**Figure 6D**), the inactivating current became apparent. At the intermediate ATP concentration of 1 μM (**Figure 6C**), hP2X4-and hP2X7-dependent currents were almost completely balanced, leading to almost constant current amplitudes over the 2–12 s time course of the ATP application.

#### Effect of P2X4 Modulators on Coexpressed hP2X4 and hP2X7 Receptor Subunits

**FIGURE 7:**
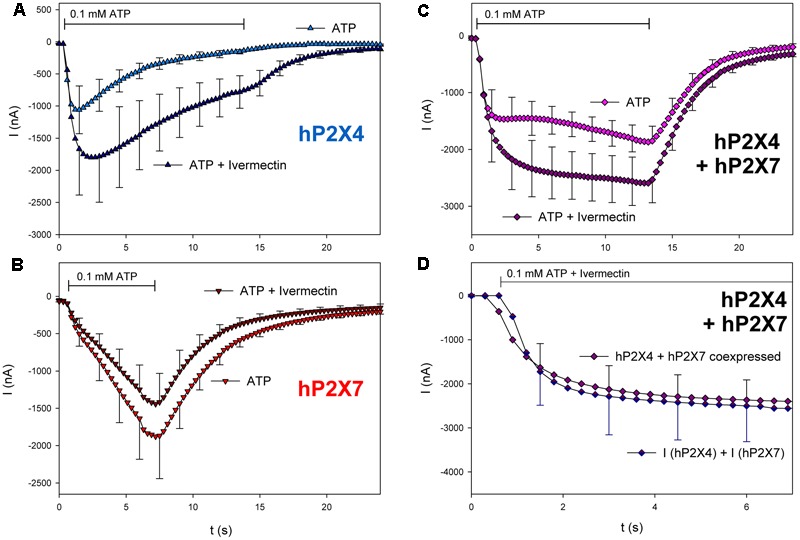
Effect of ivermectin on ATP-induced currents of hP2X4/hP2X7-coexpressing oocytes. **(A–C)** Two-electrode voltage clamp measurements showing the effect of ivermectin (3 μM) on ATP-induced currents of oocytes expressing **(A)** hP2X4, **(B)** hP2X7, or **(C)** hP2X4 + hP2X7. Oocytes were incubated for 1 min with 3 μM ivermectin, and then with 3 μM ivermectin + 0.1 mM ATP. **(D)** Current amplitudes measured in P2X4/P2X7-coexpressing oocytes during ivermectin application were compared with the sum of the P2X4- and P2X7-dependent current amplitudes (as shown in parts **A,B**, respectively). Mean current amplitudes were not significantly different at any time point, indicating the lack of a distinct P2X4/P2X7 phenotype related to ivermectin. This may be explained by ivermectin affecting only homomeric P2X4 receptors. Data are the means ± SD from 4–6 oocytes.

We next investigated the pharmacologic phenotype of coexpressed hP2X4 and hP2X7 using subunit-specific activators and inhibitors. First, we investigated the effects of ivermectin, which potentiates P2X4-dependent ATP-induced ion currents (Priel and Silberberg, 2004). As shown in **Figure 7**, coapplication of ivermectin and ATP increased hP2X4-dependent currents (**Figure 7A**) but not hP2X7-dependent currents (**Figure 7B**). Currents in hP2X4/hP2X7-coexpressing oocytes were also stimulated by ivermectin (**Figure 7C**). However, the increased current amplitude was not significantly different from the sum of hP2X4- and hP2X7-dependent currents, as measured in oocytes expressing each P2X subtype.

**FIGURE 8:**
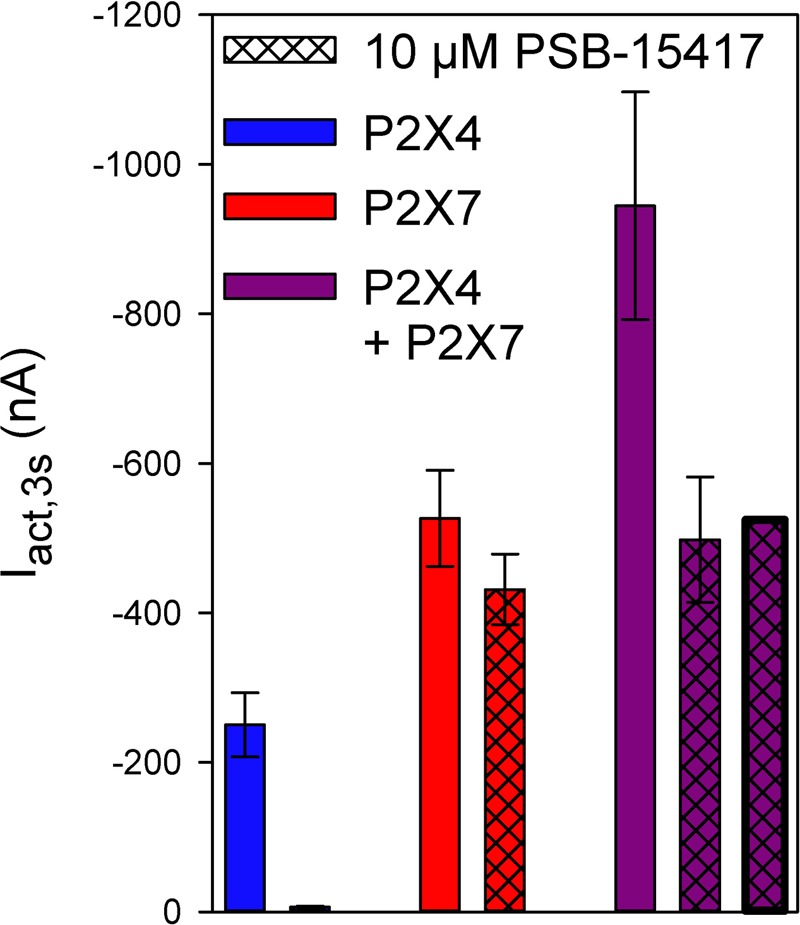
Effect of PSB-14517 on ATP-induced currents of hP2X4/hP2X7-coexpressing oocytes. Two-electrode voltage clamp measurements showing ATP-induced current amplitudes (I_act,3s_) at 3 s after the application of 0.1 mM ATP. The theoretical current remaining after PSB-14517 application to hP2X4/hP2X7-expressing oocytes (rightmost column) was calculated as described in Section “Results.” The theoretical current amplitude was not significantly different from the measured value (next to rightmost column), indicating the lack of a distinct P2X4/P2X7 phenotype related to PSB-14517, which may be explained by PSB-14517 affecting only homomeric P2X4 receptors. Data are means ± SD from 4–11 oocytes.

Next, we measured the effect of adding PSB-15417, a P2X4 receptor-selective inhibitor. The addition of 10 μM PSB-15417 completely blocked hP2X4-mediated currents (**Figure 8**, column 1 vs. column 2), whereas hP2X7-dependent currents were reduced by only 18% (**Figure 8**, column 3 vs. column 4). To calculate the extent of PSB-15417 inhibition of hP2X4/hP2X7-coexpressed subunits, we assumed that in oocytes coexpressing hP2X4 and hP2X7 (**Figure 8**, column 5) 32% of the ATP-induced current resulted from P2X4 and 68% from P2X7, based on the amplitudes measured in oocytes expressing hP2X4 (**Figure 8**, column 1) or hP2X7 (**Figure 8**, column 3) alone and the fact that hP2X7-dependent currents do not undergo desensitization (**Figure 5**). By assuming that PSB-15417 has the same effect on hP2X4- and hP2X7-mediated currents in hP2X4/hP2X7-coexpressing oocytes as in oocytes expressing hP2X4 or hP2X7 alone (i.e., reduction by 100 or 18%, respectively), a theoretical value for the current remaining after applying PSB-15417 to hP2X4/hP2X7-coexpressing oocytes was calculated (**Figure 8**, column 7). This value was not significantly different from the measured value (**Figure 8**, column 6).

#### Effect of P2X7 Agonist and Antagonists on Coexpressed hP2X4 and hP2X7 Subunits

**FIGURE 9:**
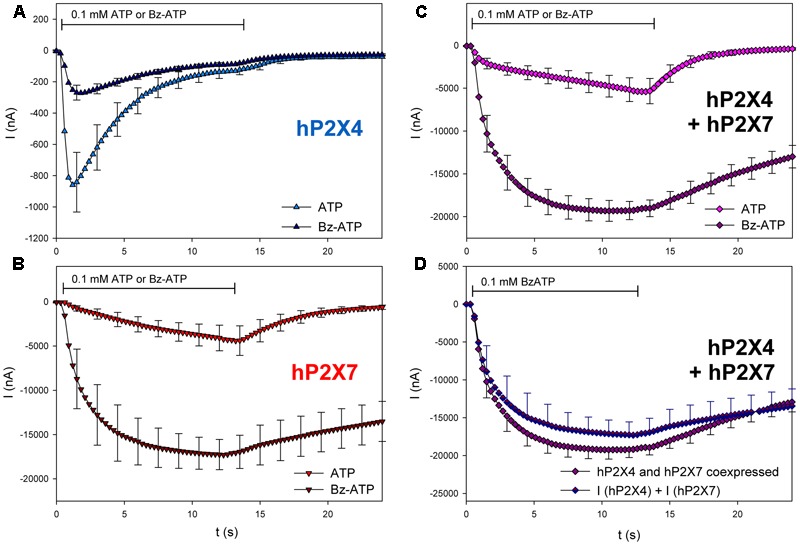
Effect of BzATP on ATP-induced currents of hP2X4/hP2X7-coexpressing oocytes. **(A–C)** Two-electrode voltage clamp measurements showing the effect of BzATP on ATP-induced currents of oocytes expressing **(A)** hP2X4, **(B)** hP2X7, or **(C)** hP2X4 + hP2X7. **(D)** BzATP-induced currents measured in P2X4/P2X7-coexpressing oocytes were compared with the sum of P2X4- and P2X7-dependent current amplitudes (shown in parts **A,B**, respectively). Mean current amplitudes were not significantly different at any time point, indicating the lack of a distinct P2X4/P2X7 phenotype related to BzATP stimulation. means ± SD from 5–6 oocytes.

Oocytes expressing hP2X4, hP2X7, and hP2X4/hP2X7 were treated with the P2X7 agonist BzATP (**Figure 9**). As we expected from previous reports (von Kügelgen, 2008), 0.1 mM BzATP was less effective against hP2X4 compared with 0.1 mM ATP (**Figure 9A**). However, it was much more effective against hP2X7 than against hP2X4 (**Figure 9B**). The amplitude of the BzATP-stimulated current in hP2X4/hP2X7-coexpressing oocytes (**Figure 9C**) was equal to the sum of the current amplitudes measured in oocytes expressing either hP2X4 or hP2X7 alone (**Figure 9D**).

**FIGURE 10:**
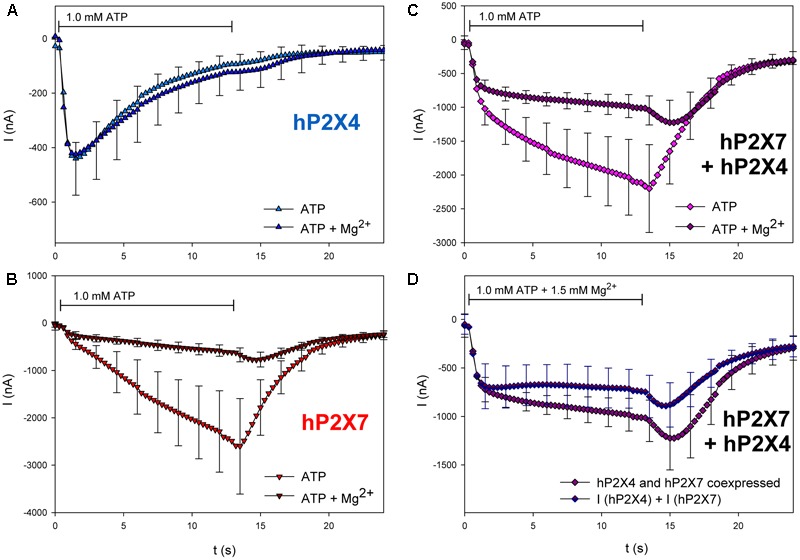
Effect of Mg^2+^ on ATP-induced currents of hP2X4/hP2X7-coexpressing oocytes. **(A–C)** Two-electrode voltage clamp measurements showing the effect of Mg^2+^ on ATP-induced currents of oocytes expressing **(A)** hP2X4, **(B)** hP2X7, or **(C)** hP2X4 + hP2X7. **(D)** Currents induced by the coapplication of ATP and 1.5 mM Mg^2+^ measured in P2X4/P2X7-coexpressing oocytes were compared with the sum of P2X4- and P2X7-dependent current amplitudes (shown in parts **A,B**, respectively). Mean current amplitudes were not significantly different at any time point, indicating the lack of a distinct P2X4/P2X7 phenotype related to Mg^2+^ inhibition. Data are means ± SD from 4–6 oocytes.

**FIGURE 11:**
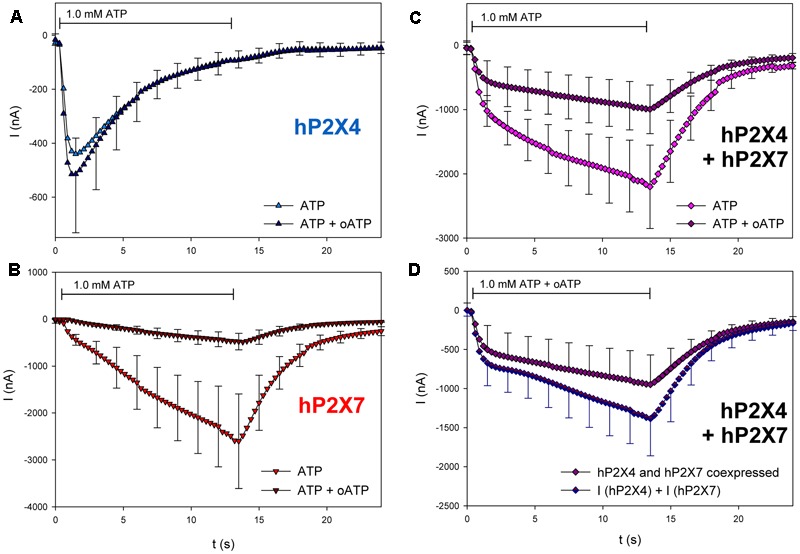
Effect of oATP on ATP-induced currents of hP2X4/hP2X7-coexpressing oocytes. **(A–C)** Two-electrode voltage clamp measurements showing the effect of 3 h preincubation in 0.3 mM oATP on ATP-induced currents of oocytes expressing **(A)** hP2X4, **(B)** hP2X7, or **(C)** hP2X4 + hP2X7. **(D)** Currents induced by ATP after oATP preincubation in P2X4/P2X7-coexpressing oocytes were compared with the sum of P2X4- and P2X7-dependent current amplitudes (shown in parts **A,B**, respectively). Mean current amplitudes were not significantly different at any time point, indicating the lack of a distinct P2X4/P2X7 phenotype related to oATP antagonism. Data are the means ± SD from 4–5 oocytes.

Next, we tested the effects of three different P2X7 antagonists/blockers on the currents in hP2X4/hP2X7-coexpressing oocytes. P2X7-dependent ion currents (North, 2002; Seyffert et al., 2004; Li et al., 2013) and Ca^2+^ signals (Pippel et al., 2015) are blocked by Mg^2+^ ions. Consistent with these findings, we found that Mg^2+^ coapplication reduces ATP-induced currents in hP2X7-expressing oocytes (**Figure 10B**) but not in those expressing hP2X4 (**Figure 10A**). The inhibitory effect of Mg^2+^ on hP2X4/hP2X7-coexpressing oocytes (**Figure 10C**) was equal to the sum of the separate effects of Mg^2+^ on hP2X4 and on hP2X7 (**Figure 10D**). The P2X7 antagonist oxidized ATP (oATP) (North, 2002; von Kügelgen, 2008) had qualitatively similar effects (**Figure 11**).

**FIGURE 12:**
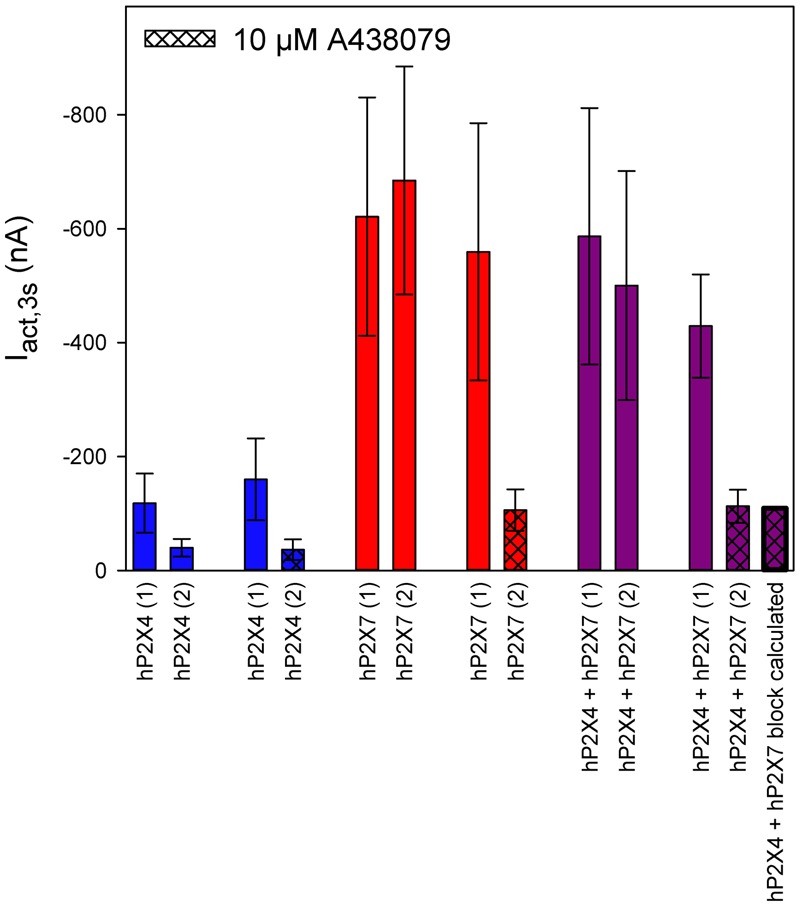
Effect of the P2X7 antagonist A438079 on ATP-induced currents of hP2X4/hP2X7-coexpressing oocytes. Two-electrode voltage clamp measurements of ATP-induced current amplitudes (I_act,3s_) at 3 s after the application of 0.1 mM ATP. Current amplitudes of the first and second ATP application are denoted (1) and (2), respectively. The theoretical current remaining after applying A438079 to P2X4/P2X7-expressing oocytes (rightmost column) was calculated as described in Section “Results.” The theoretical current amplitude was not significantly different from the measured value (next to rightmost column), indicating the lack of a P2X4/P2X7 phenotype related to A438079, which may be explained by A438079 affecting only homomeric P2X7 receptors. Data are means ± SD from 4–11 oocytes.

We investigated the effects of the P2X7-specific blocker A438079 (Nelson et al., 2006) using a protocol similar to the one used for PSB-15417 (**Figure 12**). As before, we normalized the amplitudes of the ATP-induced currents to those measured during a previous application of 0.1 mM ATP. Owing to hP2X4 desensitization, the amplitude of the second ATP-induced current was reduced by 70% on average (**Figure 12**, column 1 vs. column 2). Application of A438079 did not change this desensitization rate (**Figure 12**, column 3 vs. column 4), indicating a lack of effect on hP2X4. In contrast, P2X7-dependent currents did not display desensitization (**Figure 12**, column 5 vs. column 6). Considering 70% desensitization of hP2X4, the percentage of functional P2X4 in oocytes coexpressing P2X4 and P2X7 was calculated to be 20% (**Figure 12**, column 9 vs. column 10). By taking this into account, as well as the mean blocking effect of A438079 of 81% (**Figure 12**, column 7 vs. column 8), we calculated the ATP-induced current amplitude in hP2X4/hP2X7-coexpressing oocytes that was not blocked by A438079 (**Figure 12**, column 13). The calculated value for the amplitude was not significantly different from the measured value (**Figure 12**, column 12).

## Discussion

### FRET as a Tool to Investigate Physical Interaction of P2X4 and P2X7 Receptor Subunits

Our FRET measurements indicate a close association between P2X4 and P2X7 receptor subunits. Furthermore, FRET values were similar to those of positive controls (coexpressed homomeric GFP- and RFP-labeled P2X4 or P2X7 constructs) when the fluorophore was incorporated within the extracellular loop. Similar FE values were obtained for homomers and heteromers, indicating an abundance of P2X4/P2X7 heteromers. We were unable to study several P2X4 and P2X7 constructs in which the label was incorporated within the extracellular domain because of their weak expression. For extracellularly labeled constructs with reasonable expression levels, FEs were much larger compared with the FEs of C-terminally fluorescence-labeled constructs. The differences in FRET efficiency are consistent with a model in which the fluorophore moiety has approximately the same relative distance from the (outer) membrane surface in both ectodomain-labeled P2X4 and P2X7 proteins. In C-terminally labeled constructs, the location of the fluorophore moiety relative to the (inner) membrane surface is strongly influenced by the length of the C-terminal tail, which is much shorter in the hP2X4 subunit (29 residues) than in the hP2X7 subunit (∼300 residues; see Supplementary Figures 2–27). Therefore, FE was very low or absent when C-terminally fluorescence-labeled P2X4 and P2X7 constructs were coexpressed. In contrast, coexpression of C-terminally labeled P2X4 and truncated P2X7^1-408^ resulted in significant FE values. This indicates that the C-terminus is located far from the “trunk” of the P2X7 subunit (residues 1–408). Importantly, all of the sequence elements responsible for P2X subunit assembly are located within the ectodomain; therefore, C-terminal truncation does not affect trimerization (Duckwitz et al., 2006). We also previously reported that truncated hP2X7^1-408^ subunits can assemble into functional trimers (Becker et al., 2008).

These findings contradict a previous FRET study of cyan fluorescent protein (CFP)-and yellow fluorescent protein (YFP)-labeled P2X proteins expressed in human embryonic kidney cells, which reported a larger FE for homomeric P2X7 than for homomeric P2X4 (Young et al., 2008). The authors concluded that longer rather than shorter P2X C-termini are more likely to interact. In another study using the same expression system, the P2X4/P2X7 interaction was investigated by measuring sensitized emissions of the acceptor YFP by Spectra-FRET (Perez-Flores et al., 2015). The authors reported that a significant FE for coexpressed P2X4/P2X7 was only obtained after activating the P2X7 receptor with BzATP or high ATP concentrations. No measurable FE was obtained for truncated P2X7^1-418^ constructs,. The reason for the discrepancies between these results and ours is unclear, but might be caused by differences in the choice of the expression system and the P2X constructs.

It is possible that P2X4 and P2X7 subunits interact not only as heteromers but also as homomers. Indications of close contact of P2X4 and P2X7 homomers have been reported (Boumechache et al., 2009; Weinhold et al., 2010). In contrast, atomic force microscopy measurements only rarely found dimers of distinct homotrimeric P2X4 and P2X7 receptors (Antonio et al., 2011). Although we cannot completely rule out the possibility of self-association for homotrimeric P2X4 and P2X7 receptors, in our FRET experiments, we consider it unlikely for the following reason. The probability of non-specific FRET signals is enhanced with increasing levels of subunit expression and diminished at low expression levels. However, we did not observe such a correlation (Supplementary Figure 1). It was calculated that non-specific bystander effects occur at a fluorescence molecule density of ≥2000 molecules/μm^2^ (Clayton and Chattopadhyay, 2014). Given a surface area *A* of *Xenopus* oocytes of about 2 × 10^7^ μm^2^ (Lin-Moshier and Marchant, 2013), a single channel current amplitude *i* at -40 mV of ∼0.4 pA, an open probability *P_o_* (1 mM ATP) of about 0.2 (Riedel et al., 2007), and a maximal P2X7-mediated whole-cell current *I* of 2000 nA (**Figure 4**), the molecule density *D* = *I*/(*iPoA*) = 1.25 molecules/μm^2^ for fluorescence-labeled P2X7 receptors, or 3.75 P2X7 subunits/μm^2^, far higher than the critical expression density for bystander FRET. Furthermore, the lack of FRET signals for our negative controls argue against non-specific FRET. The same line of argument holds against a possible non-specific interaction between GFP- and RFP-labeled constructs.

### Do Coexpressed P2X4 and P2X7 Receptor Subunits Have a Distinct Electrophysiological Phenotype?

Our evidence for a physical interaction between P2X4 and P2X7 subunits within heteromers led us to investigate the functional consequences of this interaction by measuring hP2X4- and hP2X7-dependent ion currents in *Xenopus* oocytes. For this, we studied the current time course, the ATP concentration dependency of current amplitudes, and the effect of P2X4- or P2X7-specific drugs in P2X4/P2X7-coexpressing oocytes. For each parameter, the result could be explained by an additive effect of the individual hP2X4 and hP2X7 current components. Likewise, the simplest explanation for the time course of ATP-activated currents in hP2X4/hP2X7-coexpressing oocytes is that only homomeric hP2X4 and hP2X7 receptors are formed, with independent functions. Furthermore, the simplest explanation for the ATP concentration-dependence of currents in oocytes coexpressing hP2X4 and hP2X7 is that at low ATP concentrations (<10 μM) only hP2X4 receptors (with high ATP sensitivity) are activated, whereas at higher ATP concentrations additional hP2X7 receptor channels (with low ATP sensitivity) are opened. This explanation does not require the formation of P2X4/P2X7 heteromers with a distinct ATP concentration dependency. Similarly, the effects of P2X4- or P2X7-specific pharmacological agents in oocytes coexpressing hP2X4 and hP2X7 can be explained by their actions on homomers of the targeted subtype only.

The lack of a functional interaction is consistent with the functionally independent P2X4 and P2X7 phenotypes reported in rat cortical microglia (Visentin et al., 1999). In contrast, functional P2X4/P2X7 interactions have been reported in airway ciliated cells (Ma et al., 2006), macrophages (Babelova et al., 2009; Kawano et al., 2012a,b), dendritic cells (Sakaki et al., 2013), and gingival epithelial cells (Hung et al., 2013).

In the present study, it is difficult to reconcile evidence for a physical interaction in the form of heteromers, as demonstrated by the FRET measurements, with the lack of a new functional phenotype, as indicated by the electrophysiological recordings. A possible explanation is that heteromers exist, but the phenotype is either P2X4-like or P2X7-like. This might depend on the presence of a single P2X4 or P2X7 subunit driving the dominant phenotype or on which subunit is incorporated twice into the trimer. Further study into the molecular mechanism of P2X receptor function is therefore necessary.

## Author Contributions

MS, KP, and AP performed experiments and evaluated data. MK designed study, performed experiments, evaluated data and critically read the manuscript. CM provided drugs and critically read the manuscript. UB made the P2X constructs. GS designed the study, evaluated data and wrote the manuscript. FM designed the study, performed experiments, evaluated data and wrote the manuscript.

## Conflict of Interest Statement

The authors declare that the research was conducted in the absence of any commercial or financial relationships that could be construed as a potential conflict of interest.

## Footnotes

1 http://www.cbs.dtu.dk/services/SignalP

2 https://fiji.sc
