# Supplementary material for: Interaction of Purinergic P2X4 and P2X7 Receptor Subunits
Source: Front Pharmacol. 2017 Nov 22;8:860. doi: 10.3389/fphar.2017.00860 (PMC5702805; doi:10.3389/fphar.2017.00860)
Supplement: Supplementary file 1 [file Table_1.pdf]

Supplementary Table 1 – Sequences of primers used for RT-PCR amplification and subcloning of the rP2X7 DNA

Construct number	Construct name	Primer number	Orienta-tion	Primer sequence
K-0887	rP2X7	O-0551	forward	5'AAA**GACGTC**GAGCCTGTCGCCATGCCCGCTTGCTGTAGCTGGAA
K-0887	rP2X7	O-0552	reverse	5'AAA**TCTAGA**TAGTCAGTAGGGATACTTGAAGCCACTGTACTGCC

Construct and primer numbers refer to lab-internal documentation lists and are shown for eventual tracking purposes. AatII (GACGTC) and XbaI (TCTAGA) restrictions sites used for directional cloning into the pNKS2 vector are bolded and underlined.

Supplementary Table 2 – Sequences of primers used for in-frame ligation of the GFP coding sequence into the hGLYRA1 DNA 3' of the signal peptide

Construct number	Final construct name	Primer number	Orienta-tion	Primer sequence
K-1555	SP^{mut}-hGLYRA1-His	O-1699	forward	5'GAGGCTGAAGCTGCT**CATATG**GATATCA**AGCGCT**CCGCACCCAAG
K-1555	SP^{mut}-hGLYRA1-His	O-1700	reverse	5'CTTGGGTGCGG**AGCGCT**TGATATC**CATATG**AGCAGCTTCAGCCTC
K-1556	SP-GFP-hGLYRA1-His	O-1701	forward	5'AAAAA**CATATG**GTGAGCAAGGGCGAGGAG
K-1556	SP-GFP-hGLYRA1-His	O-1702	reverse	5'AAAAA**AGCGCT**TGTACAGCTCGTCCATGCCGA

Construct and primer numbers refer to lab-internal documentation lists and are shown for eventual tracking purposes. NdeI (CATATG) and Eco47IIII (AGCGCT) restrictions sites (bolded and underlined) were first inserted by QuikChange mutagenesis and then used for the directional subcloning of the PCR-amplified GFP coding sequence.

Supplementary Table 3 – Sequences of megaprimers used in this study

Construct number	Final construct name	Primer number	Orienta-tion	Megaprimer sequence
K-6530	hP2X4-GFP	O-7149	forward	5′GCAGGGTCTTGCTAGTGAGCTGGACCAG**GTGAGCAAGGGCGAGGAG**
K-6530	hP2X4-GFP	O-7150	reverse	5′GGCGCGCCCGGGAATTCCTCA**CTTGTACAGCTCGTCCATGCC**
K-6531	hP2X4-RFP	O-7697	forward	5′GCAGGGTCTTGCTAGTGAGCTGGACCAG**ATGGTGTCTAAGGGCGAAGAGC**
K-6531	hP2X4-RFP	O-7618	reverse	5′GGCGCGCCCGGGAATTCCTCA**ATTAAGTTTGTGCCCCAGTTTGC**
K-6926	hP2X4-GFP-RFP	O-9216	forward	5′**GATCACTCTCGGCATGGACGAGCTGTACAAGATGGTGTCTAAGGGCGAAGAGC**
K-6926	hP2X4-GFP-RFP	O-9278	reverse	5′CCTCGATGTTGTGGCGGATCTTGAAGTTCA**ATTAAGTTTGTGCCCCAGTTTGC**
K-6776	^{122}GFP-hP2X4	O-9083	forward	5′CCTGTGCCCCGAGATTCCAGATGCG**GTGAGCAAGGGCGAGGAGC**
K-6776	^{122}GFP-hP2X4	O-9082	reverse	5′GCAGTACAGCTGGCATCTGATTTACACACAGTGGT**CTTGTACAGCTCGTCCATGCC**
K-6925	^{122}RFP-hP2X4	O-9276	forward	5′CCTGTGCCCCGAGATTCCAGATGCG**ATGGTGTCTAAGGGCGAAGAGC**
K-6925	^{122}RFP-hP2X4	O-9277	reverse	5′GCAGTACAGCTGGCATCTGATTTACACACAGTGGT**ATTAAGTTTGTGCCCCAGTTTGC**
K-7080	rP2X4-GFP	O-9464	forward	5′GCAGGGTCTTTCGGGGGAGATGAACCAG**ATGGTGAGCAAGGGCGAGG**
K-7080	rP2X4-GFP	O-9465	reverse	5′GCGCCCGGGAATTCTAGAGGCGTCA**CTTGTACAGCTCGTCCATGCC**
K-7081	rP2X4-RFP	O-9466	forward	5′GCAGGGTCTTTCGGGGGAGATGAACCAG**ATGGTGTCTAAGGGCGAAGAGC**
K-7081	rP2X4-RFP	O-9467	reverse	5′GCGCCCGGGAATTCTAGAGGCGTCA**ATTAAGTTTGTGCCCCAGTTTGC**
K-7079	^{122}RFP-rP2X4	O-9462	forward	5′CAGACACAGAGCACCTGTCCAGAGATTCCTGATAAG**ATGGTGTCTAAGGGCGAAGAGC**
K-7079	^{122}RFP-rP2X4	O-9463	reverse	5′GTGCAGTCGGCGTCTGAATTACAAATGCTGGT**ATTAAGTTTGTGCCCCAGTTTGC**
K-7183	$^{122.GAGA}$GFP^{AGAG}-rP2X4	O-9578	forward	5′CTGTCCAGAGATTCCTGATAAGGGAGCTGGAGCT**ATGGTGAGCAAGGGCGAGG**
K-7183	$^{122.GAGA}$GFP^{AGAG}-rP2X4	O-9579	reverse	5′GTCTGAATTACAAATGCTGGTTCCAGCTCCAGC**CTTGTACAGCTCGTCCATGCC**
K-7081	$^{122.GAGA}$RFP^{AGAG}-rP2X4	O-9470	forward	5′CTGTCCAGAGATTCCTGATAAGGGAGCTGGAGCT**ATGGTGTCTAAGGGCGAAGAGC**
K-7081	$^{122.GAGA}$RFP^{AGAG}-rP2X4	O-9471	reverse	5′GTCTGAATTACAAATGCTGGTTCCAGCTCCAGC**ATTAAGTTTGTGCCCCAGTTTGC**
K-6893	hP2X7-GFP	O-9214	forward	5′GTGAAGGGCAGTACAGTGGCTTCAAGAGTCCTTAC**ATGGTGAGCAAGGGCGAGG**
K-6893	hP2X7-GFP	O-9215	reverse	5′CCGGGGATCCTCTAGAGCCTGGCTTCA**CTTGTACAGCTCGTCCATGCC**
K-6892	hP2X7-RFP	O-9212	forward	5′GAAGGGCAGTACAGTGGCTTCAAGAGTCCTTAC**ATGGTGTCTAAGGGCGAAGAGC**
K-6892	hP2X7-RFP	O-9213	reverse	5′CGGGGATCCTCTAGAGCCTGGCTTCA**ATTAAGTTTGTGCCCCAGTTTGC**
K-6894	hP2X7-GFP-RFP	O-9216	forward	5′**GATCACTCTCGGCATGGACGAGCTGTACAAGATGGTGTCTAAGGGCGAAGAGC**

K-6894	hP2X7-GFP-RFP	O-9217	reverse	5′CCGGGGATCCTCTAGAGCCTGGCTTCA**ATTAAGTTTGTGCCCCAGTTTGC**
K-6885	hP2X7$^{1-408}$-GFP	O-9203	forward	5′CATTAAAGTACGTGTCCTTTGTGGATGAATCCCAC**ATGGTGAGCAAGGGCGAGG**
K-6885	hP2X7$^{1-408}$-GFP	O-9204	reverse	5′CGGTACGTACCCGGGGATCCTCTAGACTCA**CTTGTACAGCTCGTCCATGCC**
K-6906	hP2X7$^{1-408}$-RFP	O-9238	forward	5′CATTAAAGTACGTGTCCTTTGTGGATGAATCCCAC**ATGGTGTCTAAGGGCGAAGAGC**
K-6906	hP2X7$^{1-408}$-RFP	O-9239	reverse	5′CGGTACGTACCCGGGGATCCTCTAGACTCA**ATTAAGTTTGTGCCCCAGTTTGC**
K-6945	rP2X7-GFP	O-9281	forward	5′CAGGGGCAGTACTCTGGCTTCAAGTATCCCTAC**ATGGTGAGCAAGGGCGAGG**
K-6945	rP2X7-GFP	O-9282	reverse	5′CGGTACGTACCCGGGGATCCTCTAGATAGTCA**CTTGTACAGCTCGTCCATGCCG**
K-6946	rP2X7-RFP	O-9283	forward	5′CAGGGGCAGTACTCTGGCTTCAAGTATCCCTAC**ATGGTGTCTAAGGGCGAAGAGC**
K-6946	rP2X7-RFP	O-9284	reverse	5′CGGTACGTACCCGGGGATCCTCTAGATAGTCA**ATTAAGTTTGTGCCCCAGTTTGC**
K-7016	$^{122.GAGA}$GFP^{AGAG}-rP2X7	O-9370	forward	5′GAGTATCCCAGCCGCGGAGCTGGAGCT**ATGGTGAGCAAGGGCGAGG**
K-7016	$^{122.GAGA}$GFP^{AGAG}-rP2X7	O-9371	reverse	5′CAGAATGGCACTGTTTACCTCCAGCTCCAGC**CTTGTACAGCTCGTCCATGCC**
K-5710	GluCl-GFP	O-7724	forward	5′GGACATCACCACCATCACCATCACCAT**ATGGTGAGCAAGGGCGAG**
K-5710	GluCl-GFP	O-7723	reverse	5′CACCACTTTGTACAAGAAAGCTGGGTTCA**CTTGTACAGCTCGTCCATGCC**
K-7181	hTrpV2-GFP	O-9574	forward	5′GTGCCCGTCCAGCTCCTCCAGTCCAAC**ATGGTGAGCAAGGGCGAGG**
K-7181	hTrpV2-GFP	O-9575	reverse	5′CGATATCACCACTTTGTACAAGAAAGCTGGGTCTCTCA**CTTGTACAGCTCGTCCATGCC**

Construct and primer numbers refer to lab-internal documentation lists and are shown for eventual tracking purposes. Nucleotide sequences that align to GFP, RFP and linker are colored green, red and blue, respectively.
