# Supplementary material for: Interaction of Purinergic P2X4 and P2X7 Receptor Subunits
Source: Front Pharmacol. 2017 Nov 22;8:860. doi: 10.3389/fphar.2017.00860 (PMC5702805; doi:10.3389/fphar.2017.00860)
Supplement: Supplementary file 2 [file Image_1.pdf]

Supplemental Figure 1. Dependence of FRET on expression of fluorescence-labeled P2X4 and P2X7 constructs. The linear correlation coefficients r between the mean GFP (green bars) or the mean RFP (red bars) fluorescence levels and the corresponding FRET efficiency for the same construct combinations and at the same regions of interests (ROIs) used in Fig. 3 are shown. Only ROIs with acceptor (RFP) bleaching of >30 % were used. None of the correlations reached significance indicating an independence of the FRET efficiency on the expression level of the fluorescence-labeled constructs. Means ± SEM of 16–150 ROIs from 5–20 cells.

Supplemental Figures 2–27: Topology models of the various FRET constructs. The sequenced coding regions of the various ion channel constructs were copied from the VNTi files and translated into protein sequences using the EMBOSS tool program (http://www.ebi.ac.uk/emboss/transeq/). The indicated locations of the transmembrane segments were taken from the UniProt Knowledgebase (UniProtKB) (http ://www .uniprot .org /uniprot /) on August, 2017. The topology cartoons were drawn using Protter at wlab.ethz.ch/protter/# (Omasits et al 2014). GFP and RFP residues are labeled green and red, respectively. The dark green squares in the ectodomains indicate Asp residues within the consensus sequence NXT/S for N-glycosylation. Where present, GAGA and AGAG linker sequences flanking the GFP or RFP moieties in the ectodomain are indicated in blue.

Omasits U, Ahrens CH, Müller S, Wollscheid B (2014) Protter: Interactive protein feature visualization and integration with experimental proteomic data. Bioinformatics 30: 884–886.

The UniProt Consortium (2017) UniProt: the universal protein knowledgebase. Nucleic Acids Research, 45: D158-D169. doi: 10.1093/nar/gkw1099

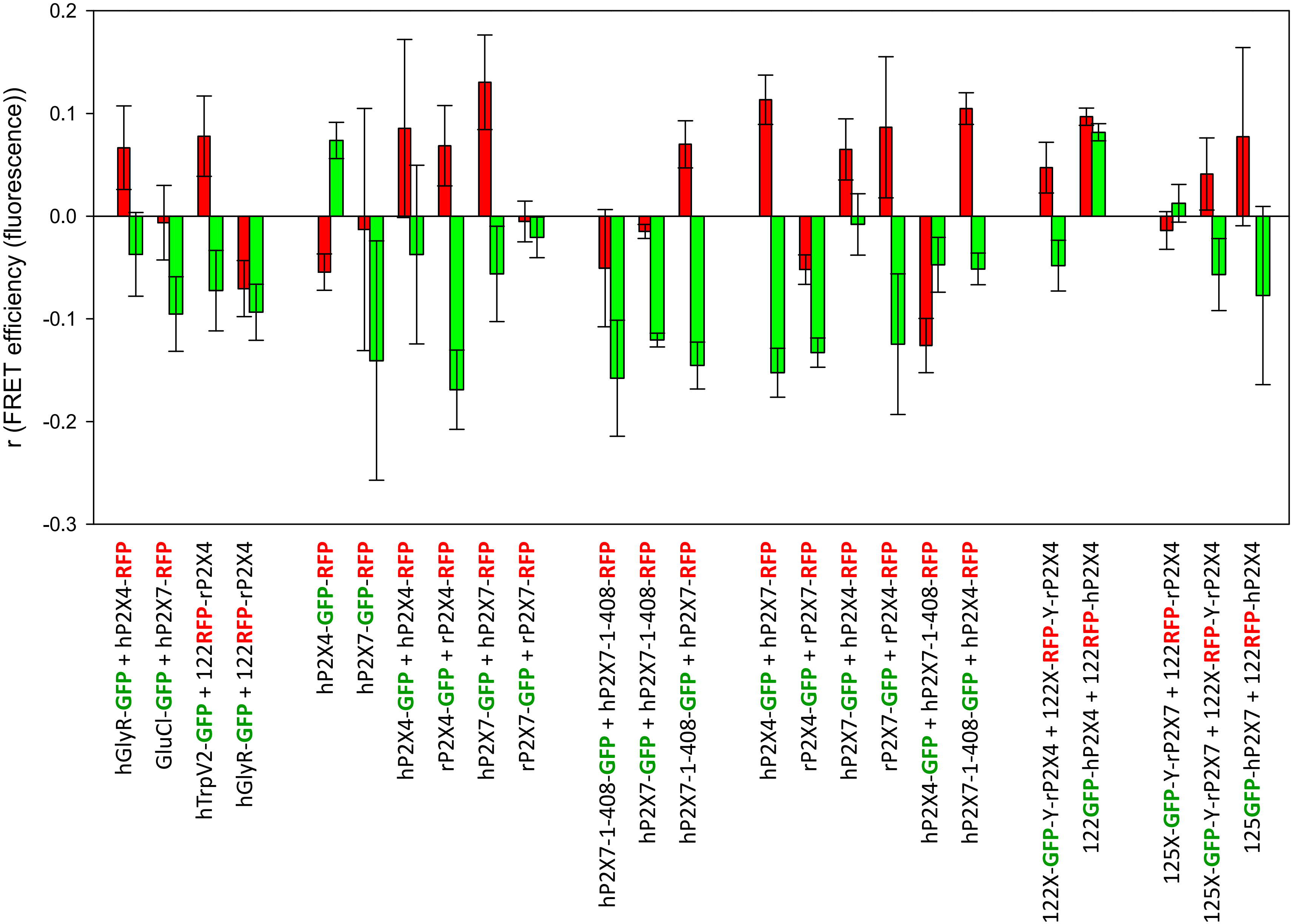

Schneider et al.
Fig. S1

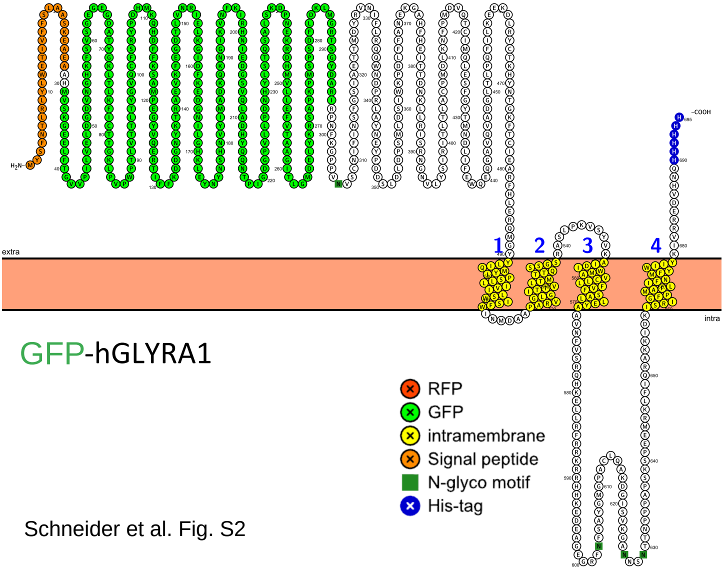

Schneider et al. Fig. S2

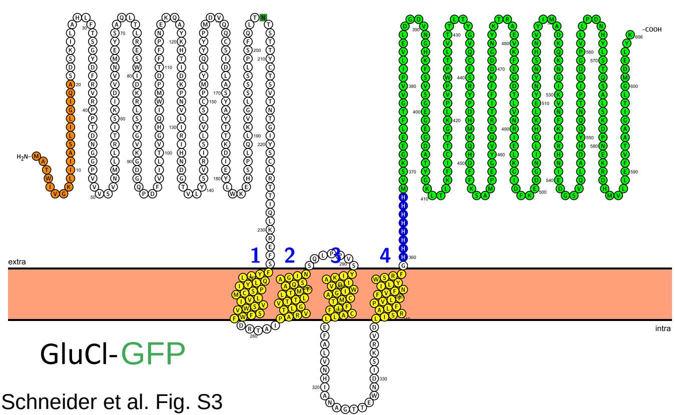

Schneider et al. Fig. S3

Schneider et al. Fig. S4

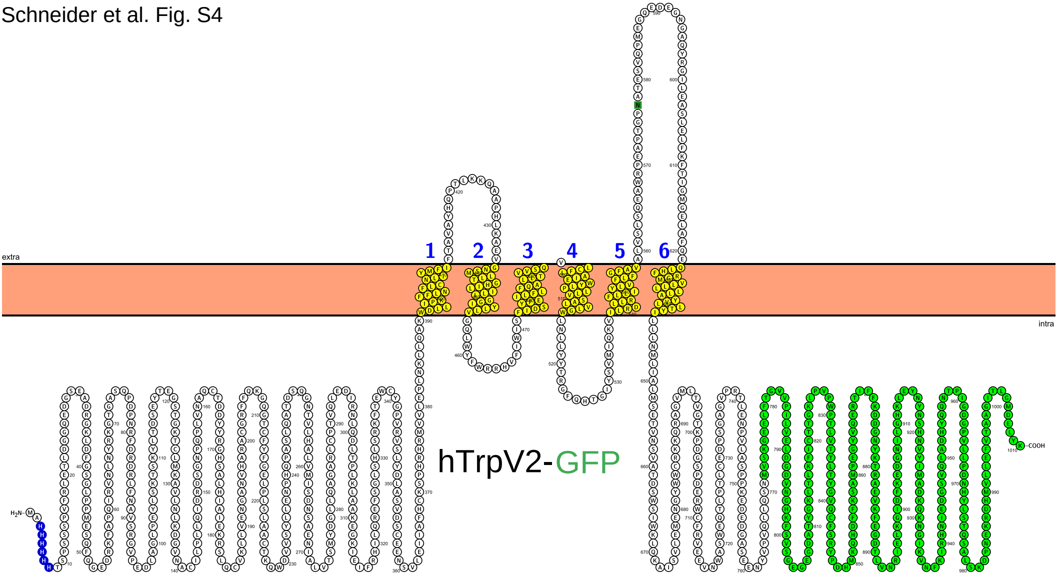

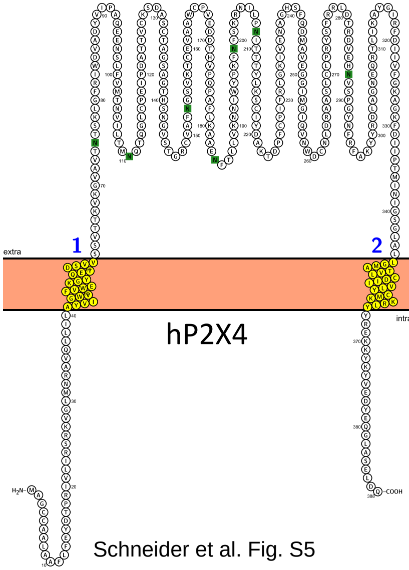

Schneider et al. Fig. S5

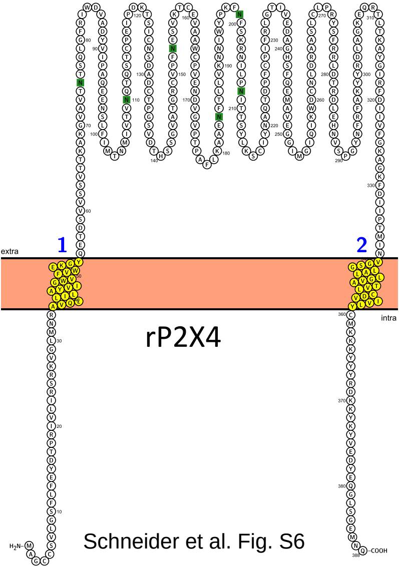

Schneider et al. Fig. S6

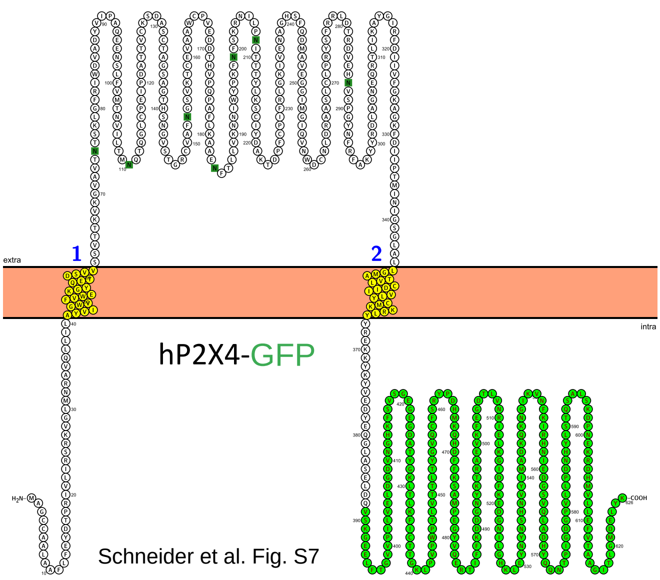

Schneider et al. Fig. S7

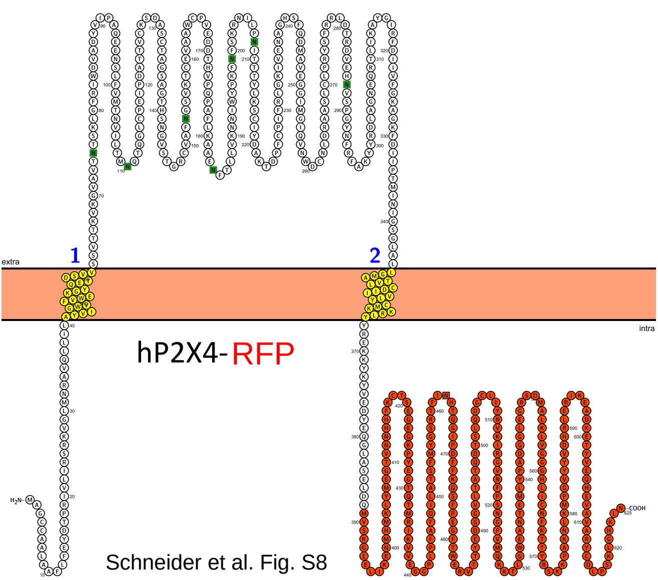

Schneider et al. Fig. S8

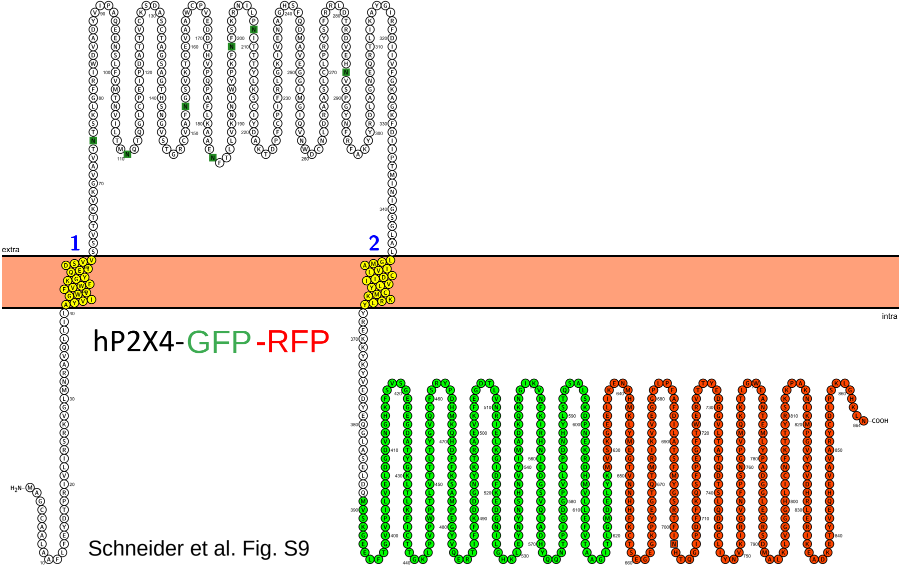

Schneider et al. Fig. S9

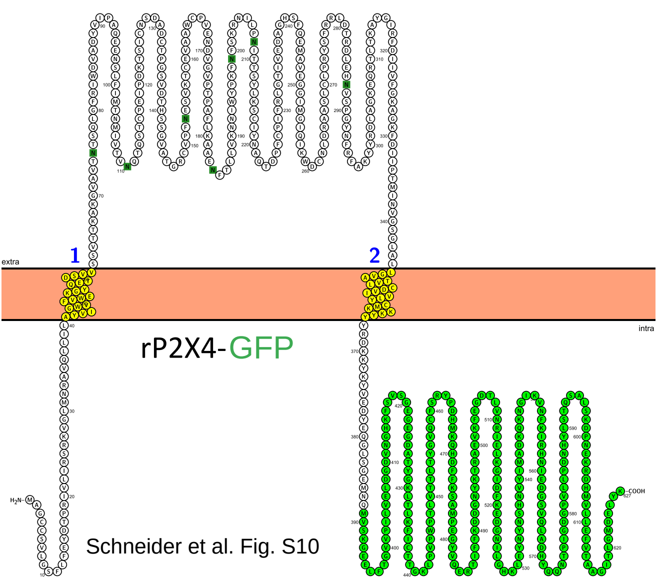

Schneider et al. Fig. S10

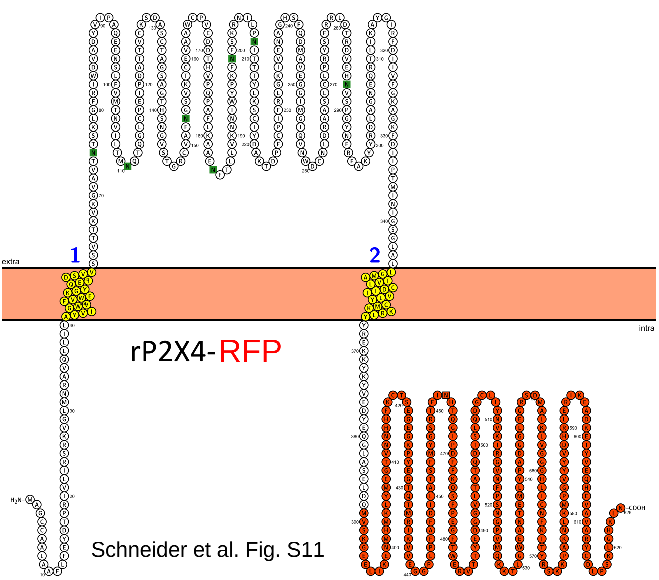

Schneider et al. Fig. S11

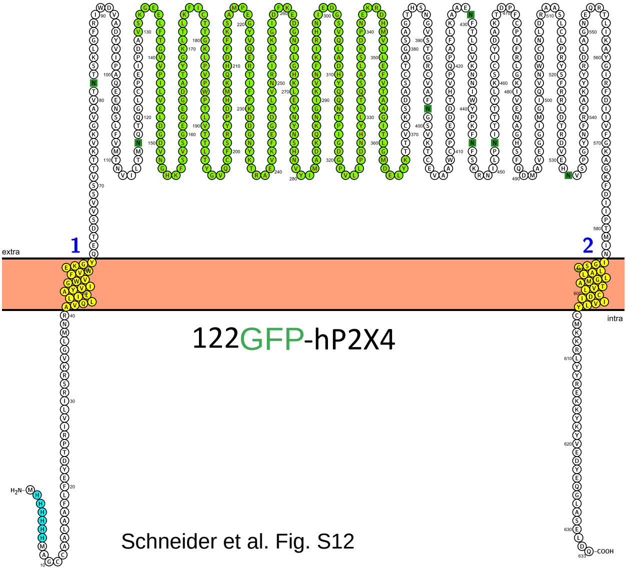

Schneider et al. Fig. S12

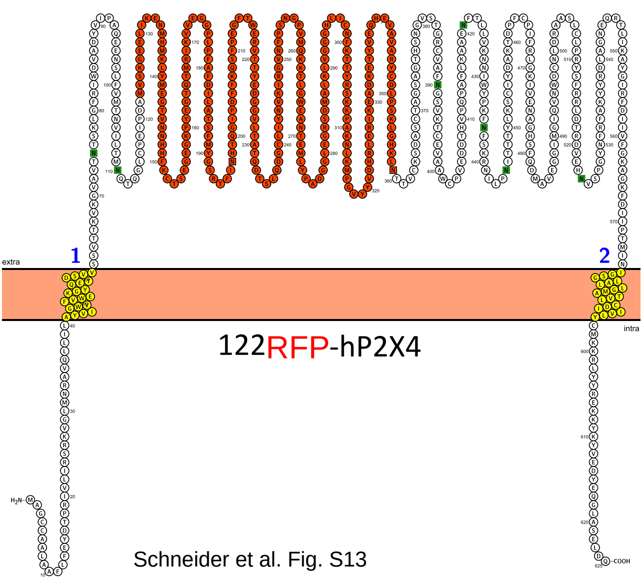

Schneider et al. Fig. S13

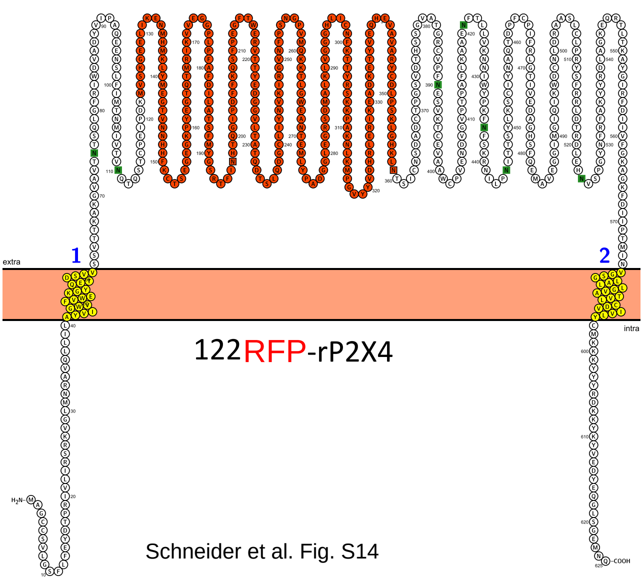

Schneider et al. Fig. S14

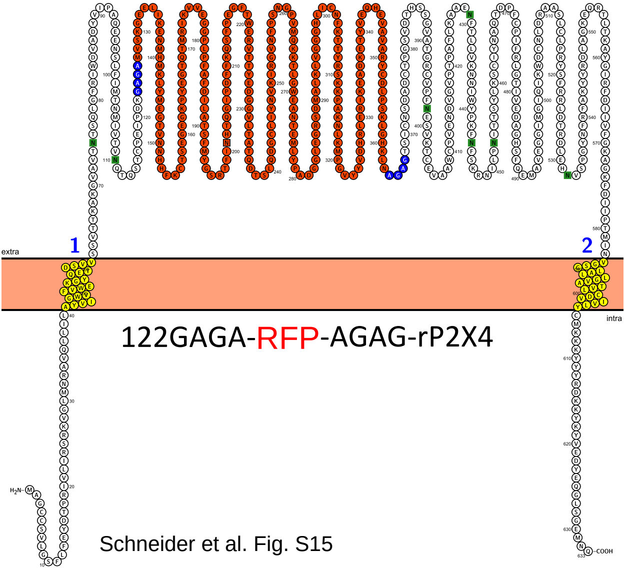

Schneider et al. Fig. S15

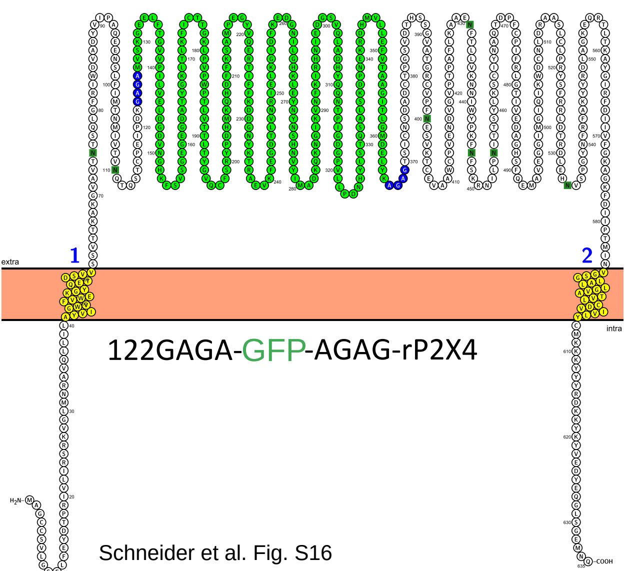

Schneider et al. Fig. S16

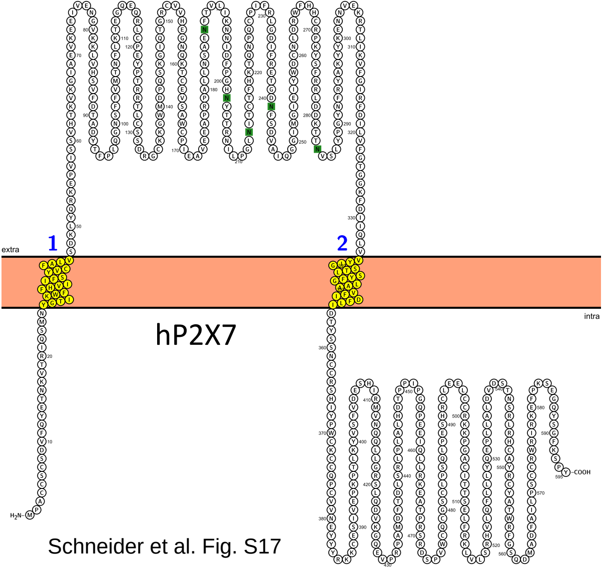

Schneider et al. Fig. S17

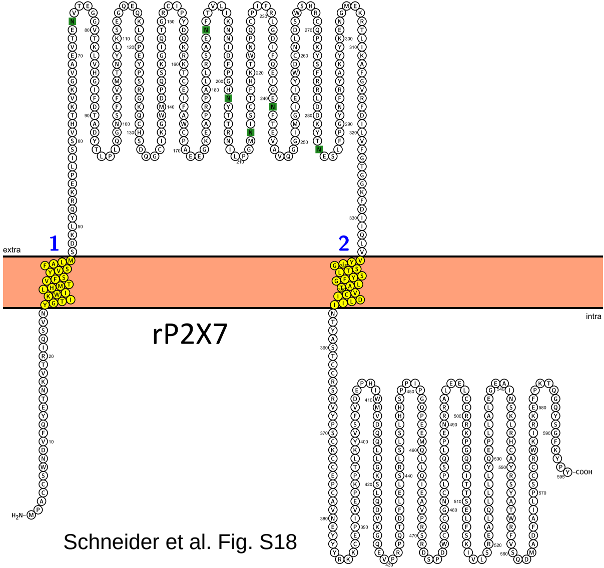

Schneider et al. Fig. S18

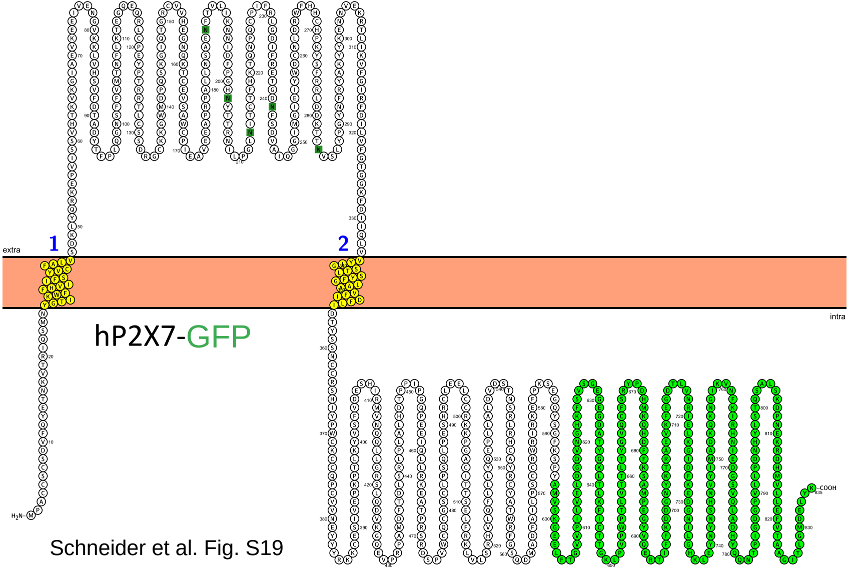

Schneider et al. Fig. S19

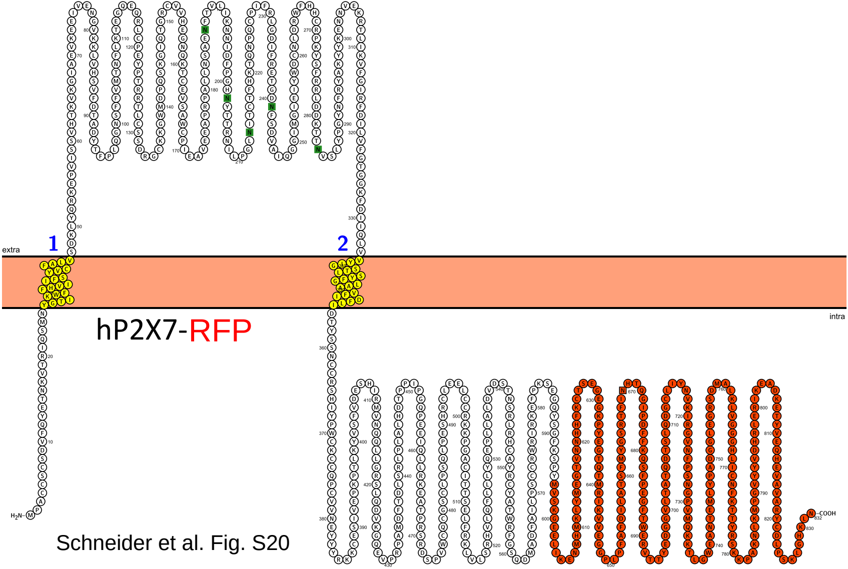

Schneider et al. Fig. S20

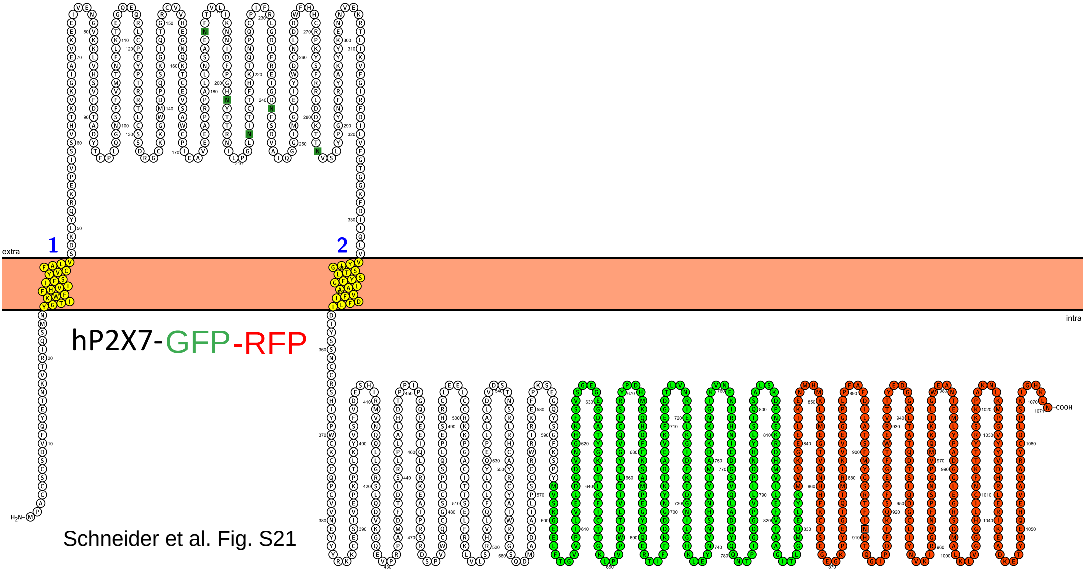

Schneider et al. Fig. S21

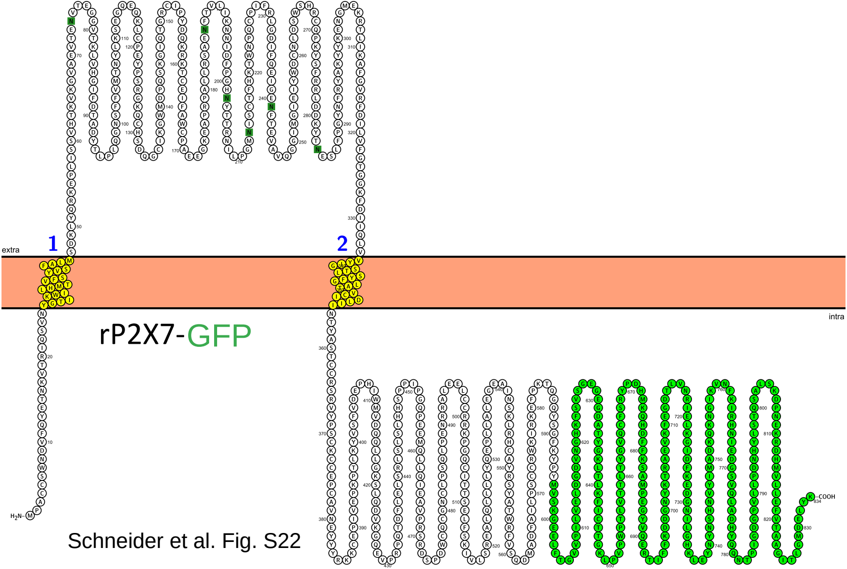

Schneider et al. Fig. S22

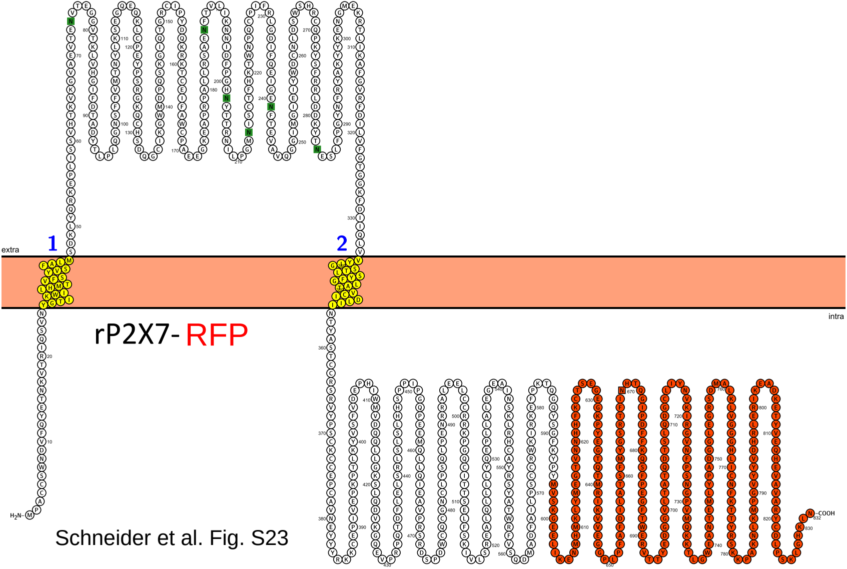

Schneider et al. Fig. S23

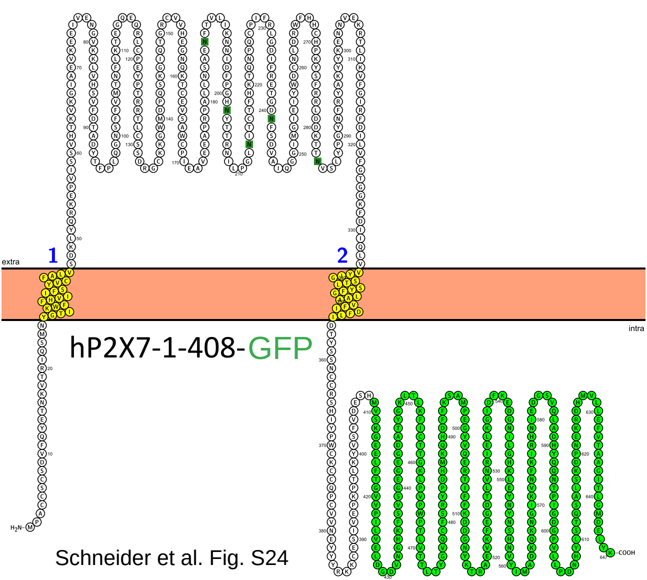

Schneider et al. Fig. S24

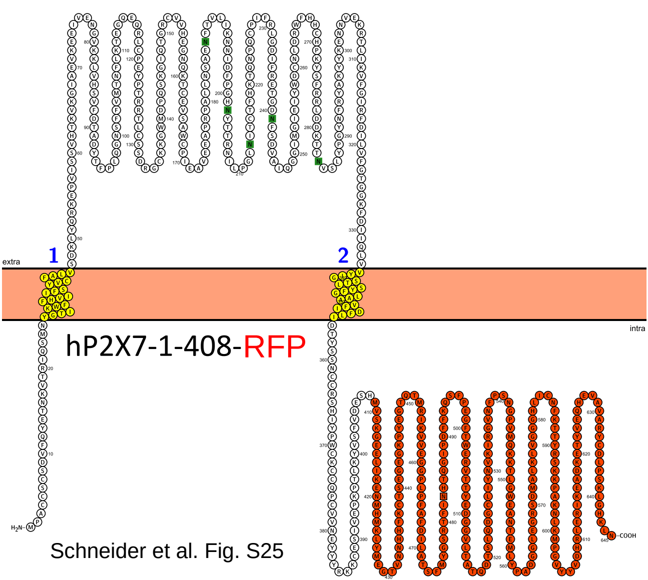

Schneider et al. Fig. S25

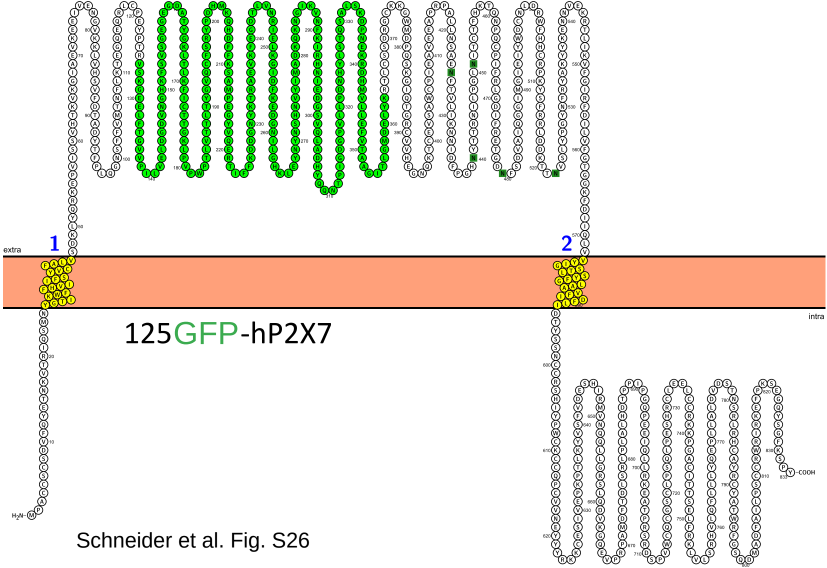

Schneider et al. Fig. S26

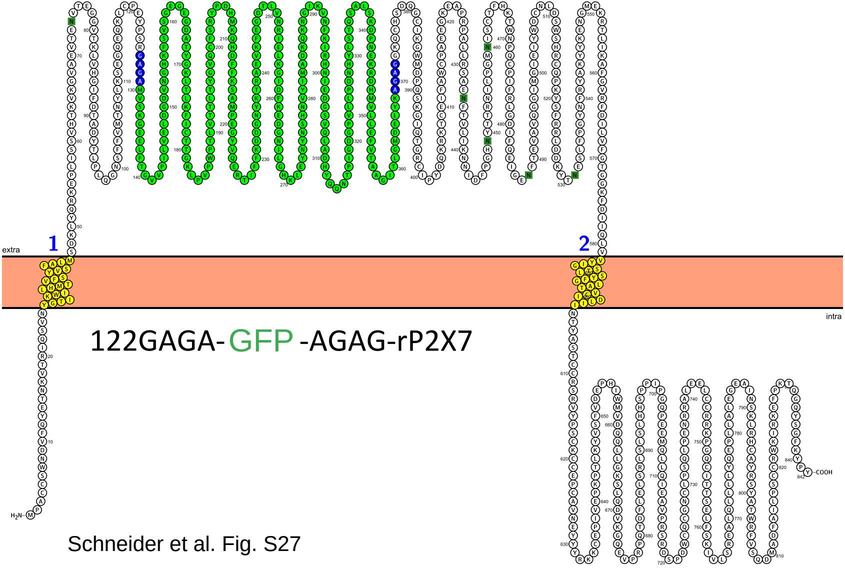

Schneider et al. Fig. S27
